# How Transcatheter Aortic Valve Implantation (TAVI) Was Born: The Struggle for a New Invention

**DOI:** 10.3389/fcvm.2021.722693

**Published:** 2021-09-29

**Authors:** Henning Rud Andersen

**Affiliations:** Department of Cardiology, Research Unit, Aarhus University Hospital, Aarhus, Denmark

**Keywords:** transcatheter, aortic stenosis, invention, development, history, patent, TAVI, TAVR

## Abstract

This story is about the invention of transcatheter aortic valve implantation (TAVI), and the people who transformed it from a concept and primitive device to a breakthrough lifesaving treatment for hundreds of thousands of patients with aortic valve stenosis. It is an inspirational example of a new disruptive technology that began with an idea most dismissed. The story describes the ups and downs from idea, design, construction, animal testing, proof-of-concept, scientific publication hurdles, a patent, license agreement, cooperation with several companies, fighting in patent courts in Europe and USA and finally how multinational companies financially bypassed the inventor. It is also a story about the struggles and battles the inventor experienced when injected into a world of lawyers and patent fights. I hope my personal story and journey can provide an inspiration and word of caution for new inventors.

## Background and History

This history leads back to Dr. Charles Dotter, the “Father of Interventional Radiology.” He invented percutaneous transluminal angioplasty and treated the first patient with a tight arterial stenosis in a leg by dilating it with tapered 8 and 12 Fr. catheters. It was in 1964 in Oregon, USA. Later that year, Dotter conducted a lecture on angioplasty in Frankfurt, Germany which was attended by Dr. Andreas Grüntzig. He was inspired by Dotter and Grüntzig conceptualized constructing the new balloon dilatation catheter and he became a pioneering inventor of Percutaneous Transluminal Coronary Angioplasty (PTCA). Grüntzig performed the first-in-man (FIM) PTCA in 1977 in Zurich, Switzerland. Both Charles Dotter and Andreas Grüntzig received a nomination for the Nobel Prize in Physiology or Medicine in 1978 for one of the most successful examples of translational medicine in the twentieth century. Shortly thereafter, Dr. Julio Palmaz was inspired by Andreas Grüntzig during a lecture in New Orleans in 1978 and went on to invent the first balloon expandable coronary stent.

## The IDEA

TAVI sprang to life in February 1989 when I concepted implanting heart valves percutaneously by catheter technique without surgery. I got my inspiration from listening to Julio Palmaz during a 1989 conference in Scottsdale, Arizona, USA. Palmaz was lecturing about how he invented and implanted balloon expandable coronary stents in animals. While I was listening, I suddenly got the idea of making the stent diameter much bigger and insert a collapsible biological valve inside the big stent. This should enable me to implant artificial heart valves using the same balloon technique described by Grüntzig and Palmaz without surgery. I was very exhilarated with my new idea. I wanted to be the first in the world to implant heart valves without heart surgery. And so, on my flight back to Denmark I formulated five requirements for the method. It should be:

- performed by retrograde catheterization
- a closed chest procedure
- a closed heart procedure
- a beating heart procedure
- performed without cardiopulmonary bypass.

## First Prototypes

**Figure 1:**
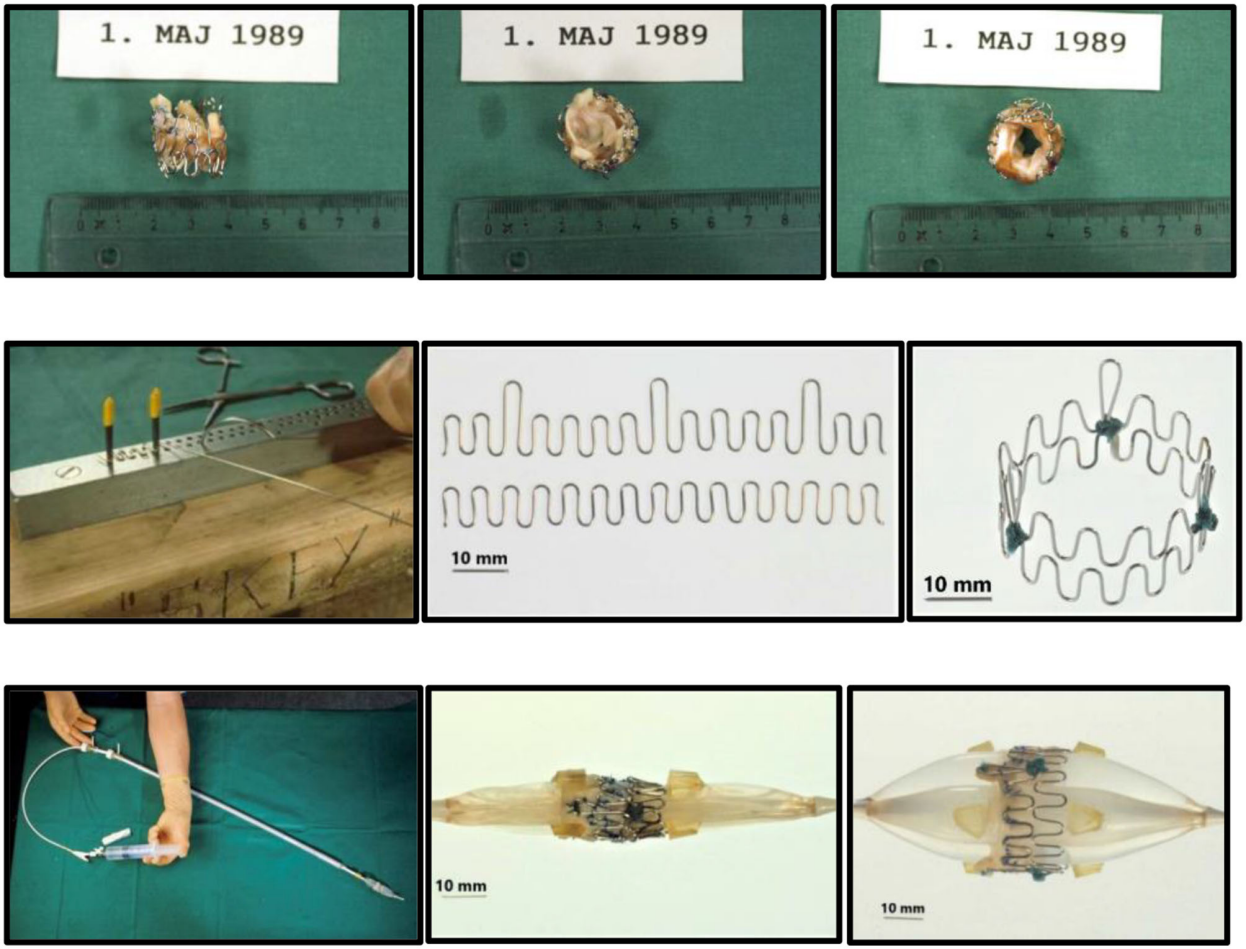
Prototype of TAVI valve and catheter technology. **Top:** The first-in-animal (FIA) valve implanted May 1. 1989. **Middle:** Later refinement of stent construction. **Bottom:** The 75 cm long, 41 Fr. introducer sheath with crimped and dilated TAVI valve on a three-foiled balloon aortic valvuloplasty dilatation catheter.

**Figure 2:**
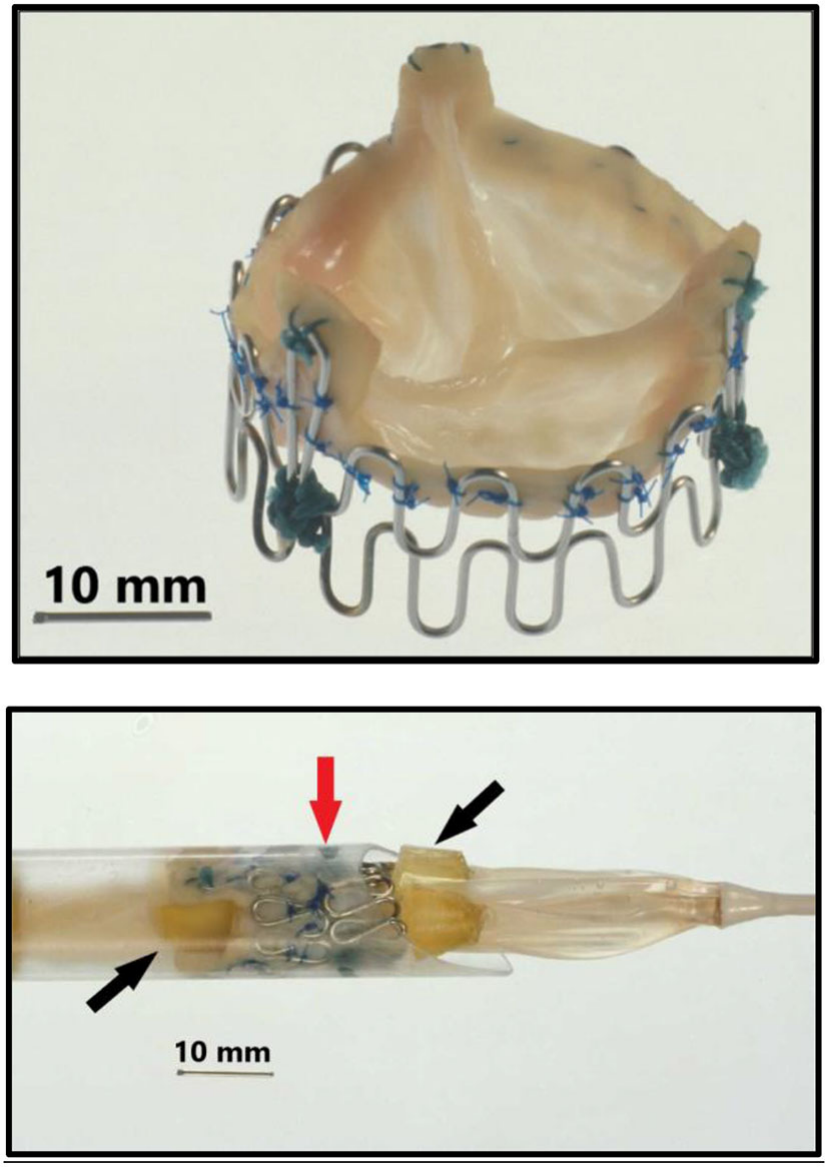
**Top:** Second generation three-leaflet handmade porcine TAVI valve with high loops for the commissure posts. **Bottom:** Tip of the 41 Fr. introducer sheath with balloon catheter inside and a crimped TAVI valve (vertical red arrow) on the middle of the balloon. Two soft silicone blocks (skewed black arrows) with a height of 3 mm were glued on each of the three balloons on the three-foiled balloon catheter. They were separated by a distance of 18 mm. The soft silicone blocks prevented the valve from sliding from the middle of the balloon during intravascular advancement and implantation.

Back in Denmark I immediately wanted to get to work, so there was no time to seek support from industry, engineers, or funding. To build stents, I bought different wires made of iron and steel from the local hardware store and procured surgical stainless-steel wires from the hospital. Initially, the various wires were bent into 15–16 loops and formed into a circle ~25 mm in diameter which was closed end-to-end by soldering. Several wires with varying thickness and stiffness were tested. The thicker wires were too stiff for balloon dilatation and the thinner too soft to maintain architectural integrity of the device. The evaluation of these mechanical parameters was done by simple visual observation and gentle finger compressions without exact measurements. I found that the surgical steel monofilament wires with a diameter of 0.55 mm fulfilled my criteria which were minimum 90% stent diameter after balloon dilatation compared to maximum balloon diameter and <10% recoil after balloon deflation followed by gentle finger compression. I soon learned that one ring did not adequately support the valve. Therefore, I tested two and three rings tied together on top of each other and found that three rings were best. Consequently, in the first-in-animal (FIA) implantation on May 18, 1989 I used three rings (Figure 1, top row). It turned out however that three rings were a bit too stiff when the valve was mounted inside as it created a small waist on the middle of the balloon during balloon dilatation. Therefore, for the succeeding experiments in 1989–1992, two rings were used. Initially, these early first-generation stents were finger folded using simple handheld tools from the hardware shop. This resulted in rather irregular bending of the loops. Afterwards, in the second-generation stents, an iron bar with holes and pins was used to make the folding much more regular (Figure 1, middle row). The first-generation stents were not constructed with three high loops for the trileaflet valves' commissure posts. Later, a young doctor under training for cardiac surgery, J. Michael Hasenkam, recommended making the stent with three high loops for the commissure posts (Figure 1, middle row and Figure 2). The biological valves were obtained from pig hearts which I bought from the local slaughterhouse. The aortic valve was carefully cut out and mounted inside the stent (Figure 2).

**Figure 3:**
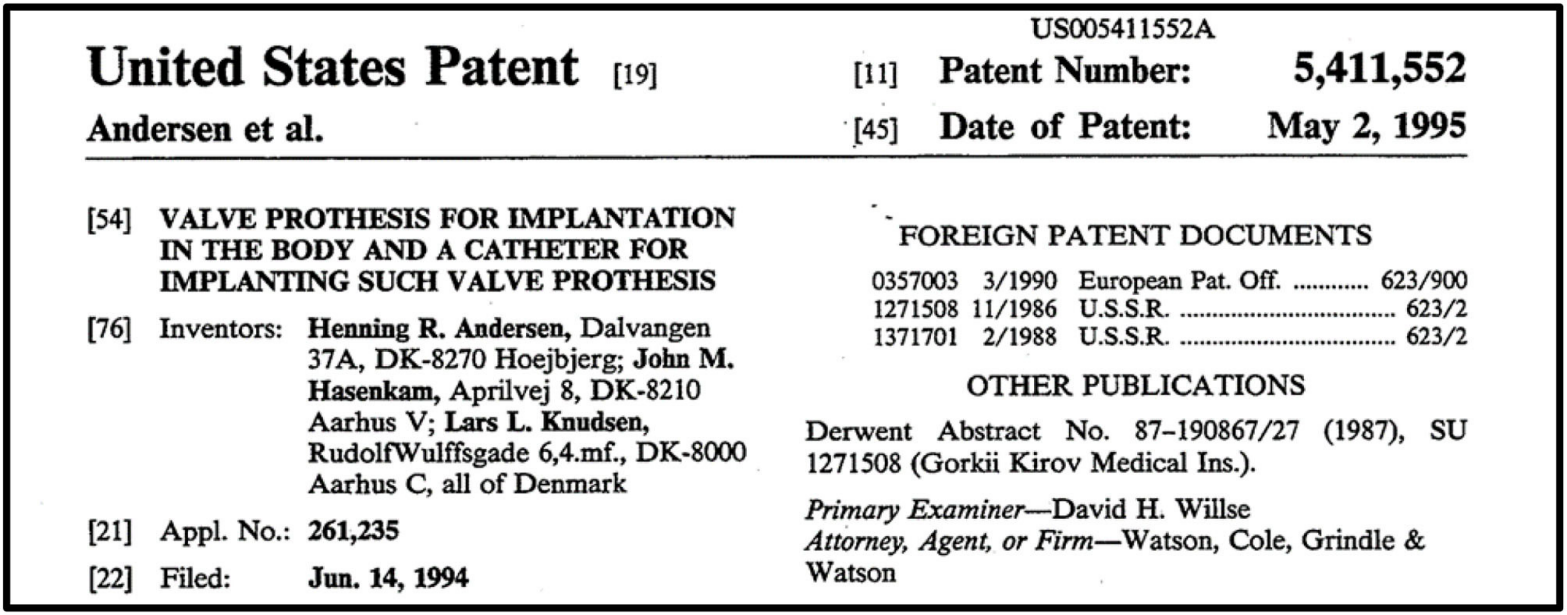
The Andersen patent. United States Patent Number 5,411,552.

Also, I was assisted by a medical student, Lars Lyhne Knudsen, to build the stents and mount the valves inside the stents. Both assisted me with the experiments and animal implantations. Therefore, I granted 25% of my patent to them to share, so we were three patent owners (Figure 3).

In the animal laboratory, I also got help from an anesthesiologist and a doctor specialized in echocardiography. Thus, the multidisciplinary TAVI heart team approach was established in the animal laboratory already when TAVI was born in 1989.

Next a 75 cm long, 41 Fr. introducer sheath with an external diameter of 13.6 mm was constructed from two flexible plastic tubes telescoping one into the other (Figure 1, bottom row, left). A reused 12 Fr. three-foiled balloon aortic valvuloplasty (BAV) dilatation catheter telescoped inside the inner plastic tube. At the tip of the sheath, a stiff plastic tube was glued which housed the balloon and the finger crimped valve during vascular introduction (Figure 2). The inner plastic tube was used to push the balloon with the crimped valve out from the stiff plastic tube.

**Figure 4:**
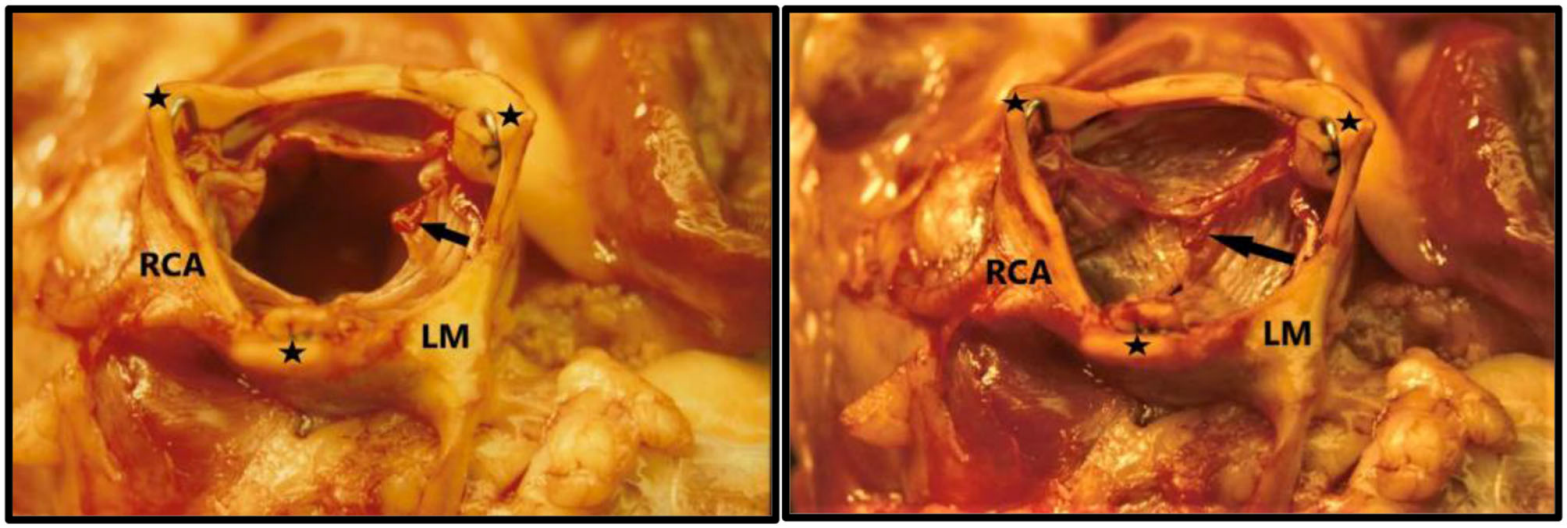
TAVI valve implanted in the sub-coronary position in 1989. The aorta is nearly tri-angular as a result of the three small valvuloplasty balloons used for dilatation and implantation. The ostia of the left main coronary artery (LM) and right coronary artery RCA) are seen. **Left:** The leaflets are open like in systole. **Right:** leaflets are closed like in diastole. The three commissure supporting posts are indicated with black stars. Small thrombi (arrows) are seen on the leaflets because the pigs were not anticoagulated during the extensive abdominal surgery and experiment due to the high risk of bleeding.

Due to my economic constraints, only a limited number of balloon catheters of random diameters were available after being used in patients. Unfortunately, they did not always match the size of the aortic annulus in the animal. They also contained X-ray contrast from their previous clinical use, which made the catheter and the balloon stiff and fragile. Furthermore, the catheters were not constructed with one circular balloon. Instead, the balloon catheters used in my institution were built with three individual longitudinal balloons (Figure 1, bottom row, right). Each of the three balloons were 70 mm long and had a diameter of 12–15 mm. Thus, the TAVI valves were not completely circular when dilated and implanted, but somewhat tri-angular in shape (Figure 4) reflecting the three small balloons. This resulted in increased paravalvular leak but they were the only balloon catheters available to me.

## First Implantation. Proof-of-Concept

**Figure 5:**
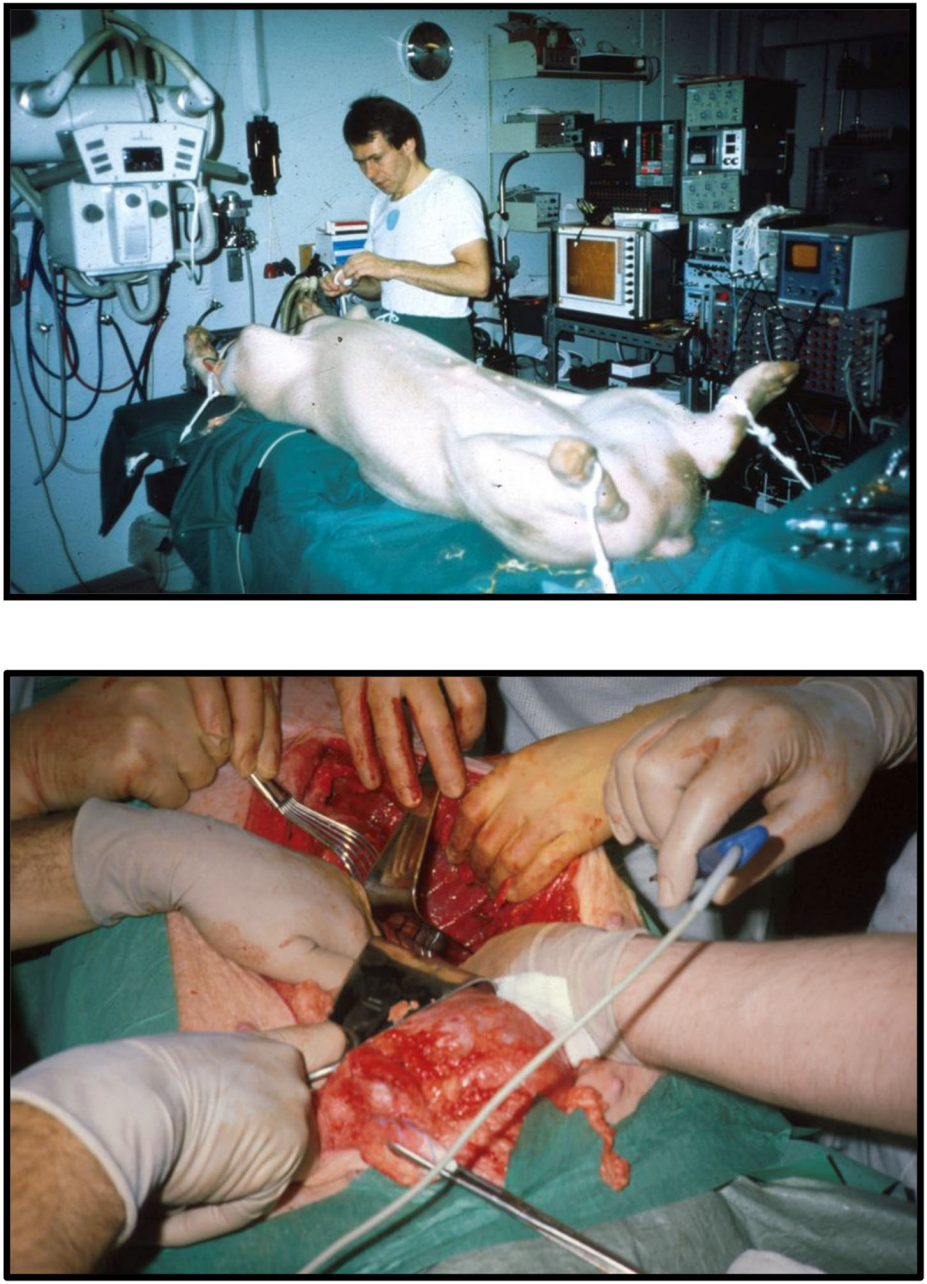
**Top:** Henning Rud Andersen preparing an 80 kg pig for TAVI implantation in 1989. **Bottom:** Minimal invasive TAVI!! To get access to the abdominal aorta and obtain enough space to sew the large vascular graft end-to-side to the aorta, the abdomen was opened through a long laparotomy. The aorta is located very deep in the retroperitoneal space in front of the spine. Therefore, the two kidneys, the spleen, the bowel and most of the intestine was removed. It generated enough space to sew the large graft end-to-side to the aorta and insert the huge 75 cm long, 41 Fr. introducer sheath retrograde into the abdominal aorta. The 8 hands on the photo illustrate the importance of a good collaboration in our TAVI heart team in 1989.

We used adult pigs for implantations (Figure 5, top). Since the femoral arteries of the pigs were only 3–4 mm in diameter, retroperitoneal access to the abdominal aorta was established (Figure 5, bottom) and a large vascular graft was sewn end-to-side to the aorta at a 45° angle. Then, the 75 cm long introducer sheath was inserted retrogradely *via* the graft into the aorta. The first implantation was performed in an 80 kg pig on May 1, 1989 (Figure 1). Luck was with us and it was a success. The time from conception of the idea in Scottsdale to initial proof-of-concept took only 2½ months.

## Refining the Technique

After the first successful procedure, we performed a series of implantations. All procedures were acute feasibility studies with animals euthanized after completion of the procedure and the heart explanted to allow inspection. Initially, not all attempts met with success. Sometimes the pig died before catheterization was initiated because of fatal bleeding caused by the extensive abdominal surgery (Figure 5, bottom). Occasionally the stiff secondhand balloons ruptured before full inflation. Sometimes, the coronary ostia were occluded. Other times the valve dislodged and embolized because the available balloons were smaller than the pig's aortic annulus. On occasion the inflated balloon with the dilated valve was pushed downstream into the ascending aorta by the blood flow. Indeed, we learned the hard way that we had to buy smaller pigs if we only had 25 mm balloons available that day. The lesson being “one size of pig does not fit all secondhand balloon catheters”! In one case, the assisting medical student mounted the valve upside down. This valve was implanted successfully in the correct location under the coronary ostia. Initially we were happy with the implantation based on the aortic angiogram which showed brisk flow into both coronary arteries. Yet happiness did not last long as we soon discovered that the valve completely blocked the blood flow from the heart. I learned that I had to double-check the valve's orientation on the balloon myself, and the young medical student was seriously advised not to turn the valve upside down again!

**Figure 6:**
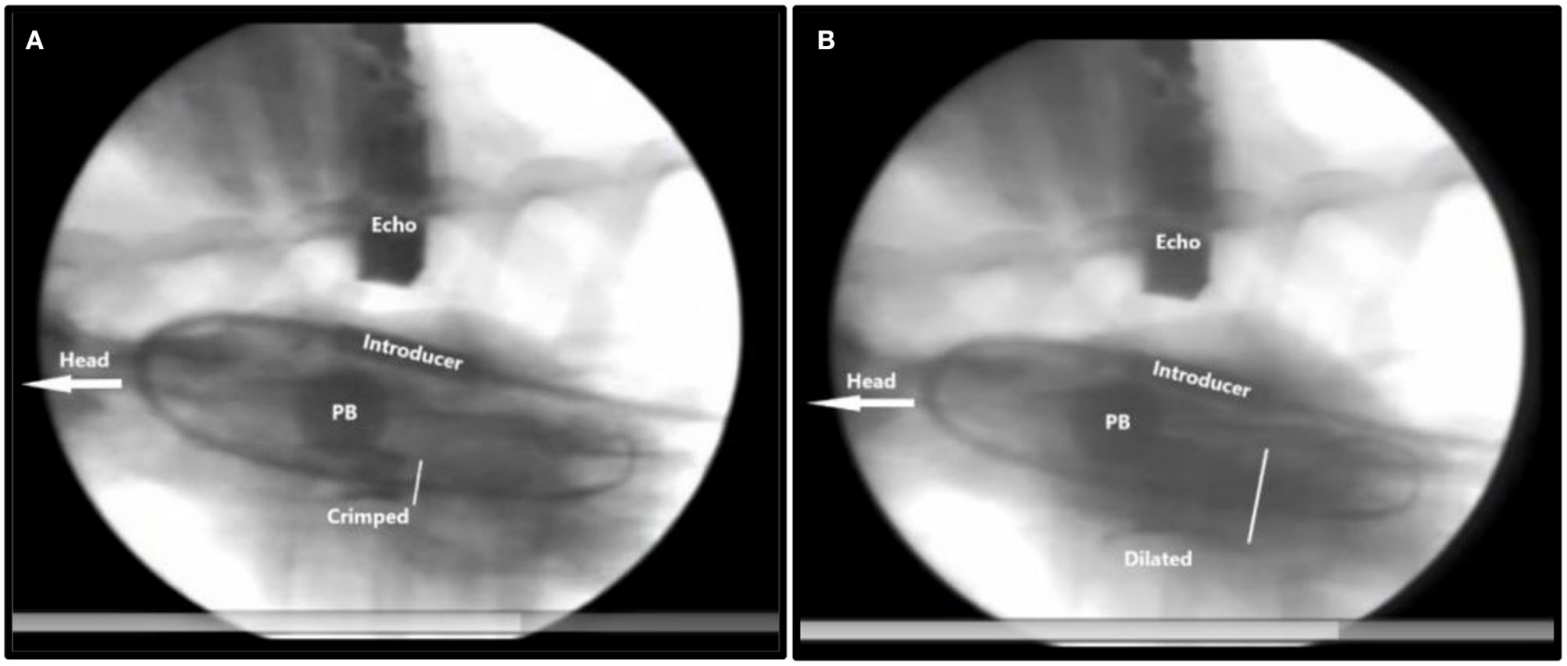
X-ray of TAVI implantation in a pig. The head of the animal is to the left. Inflated pulmonary balloon (PB) occluding the pulmonary artery trunk stopping blood flow toward the lungs and the heart. **(A)** Crimped = The crimped TAVI valve in the aortic annulus. **(B)** Dilated = The balloon dilated TAVI valve. The white bar measures the diameter of the TAVI valve. Introducer = the 41Fr. introducer sheath. Echo = transthoracic echo transducer.

I also realized that we had to temporarily stop the blood flow through the left ventricle to reduce risk of distal embolization with the blood flow during deployment. Therefore, a new experimental catheter technique was developed. Two soft 12 Fr. urine bladder catheters with a 40 mm inflatable balloon at the tip were spliced together. Then, using right heart catheterization and Swan-Ganz catheter technique, this catheter was inserted and floated into the common pulmonary trunk with the balloon inflated to only a small diameter. By inflating the balloon further to 40 mm, it blocked the pulmonary trunk preventing the blood flow toward the lungs and heart. After few seconds, the pig had a beating heart in sinus rhythm but without blood flow. We could then implant the valve without risk of distal embolization (Figure 6).

**Figure 7:**
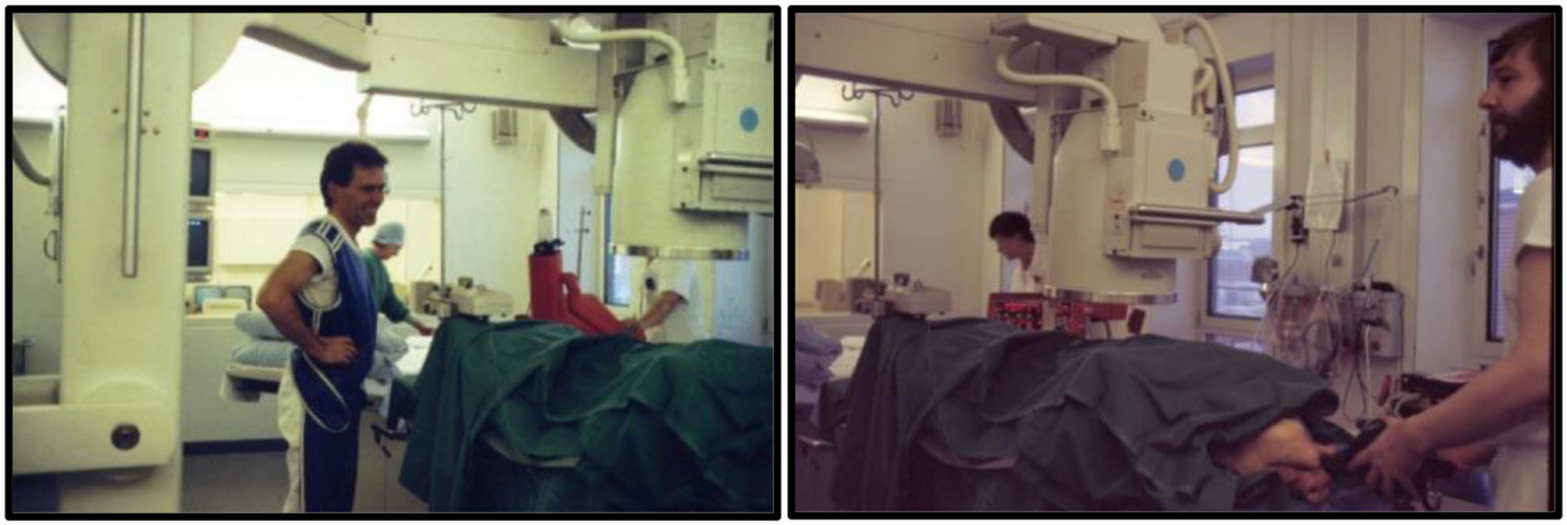
TAVI pig in the clinical cardiac catheterization laboratory after all the patients had left, 1989. The X-ray equipment was much better compared with the equipment in the experimental animal laboratory. **Left:** Henning Rud Andersen. **Right:** J. Michael Hasenkam manually ventilating the pig during the visit in the laboratory.

Ultimately, all of these various implantation challenges were encountered, understood and mitigated. These are still relevant concerns even today, all of which were seen in our early work. Most of the implantations were performed in the experimental animal laboratory, but sometimes we snuck into the clinical cardiology catheterization laboratory in the evening when the patients had left because the X-ray quality there was much better (Figure 7).

## Aortic Insufficiency Experiments

In addition to the work regarding aortic valve stenosis, a new experimental model for aortic insufficiency was developed. The purpose was to study how the TAVI valve could protect the left ventricle when it was implanted in the proximal descending thoracic aorta in pigs with severe aortic insufficiency. It mimicked the very first human surgical heart valve implantations dating back to 1952 when Dr. Charles Hufnagel performed the first insertion of caged-ball valves in the proximal descending thoracic aorta in patients with native aortic valve insufficiency (1, 2). First, the pigs were opened through a long laparotomy (Figure 5) which continued through a long sternum split which nearly divided the animal into two halves. An electromagnetic flow probe was then mounted on the ascending aorta, and pigtail catheters were inserted from the carotid arteries into the left ventricle and aorta. Baseline hemodynamic and angiographic measurements were performed. Then, the huge 75 cm long 41 Fr. introducer sheath was inserted retrograde *via* the graft into the abdominal aorta, and the TAVI valve was implanted in the proximal part of the descending thoracic aorta. To create aortic incompetence, a plastic tube with an inner cross-sectional area of 100 mm^2^ and with multiple side holes was inserted retrograde in the ascending aorta and placed across the native aortic valve. It created severe acute aortic valve regurgitation. New measurements and angiograms were then performed. Initially, the TAVI valve in the proximal descending thoracic aorta blocked the blood volume from the lower part of the body, 80–85% of total blood volume, to regurgitate. Then, only the blood from the head and the front legs, 15–20% of total blood volume, could regurgitate. Thereby, the TAVI valve partially “protected” the heart with the acutely insufficient native valve and resulted in only moderate aortic insufficiency. Afterwards, a special homemade catheter was inserted from the carotid artery into the TAVI valve. The catheter could push aside the three leaflets eliminating the function of the TAVI valve. It allowed the blood volume from the lower part of the body to regurgitate back through the TAVI valve toward the heart, thus creating acute, severe aortic insufficiency. Measurements were performed again showing increased end-diastolic pressure in the severely insufficient, “unprotected” left ventricle (Table 1).

**Table 1:** Left ventricular blood pressure (median values, *n* = 6).

Baseline	120/8 mmHg
Aortic insufficiency with intact TAVI valve function	88/29 mmHg
Aortic insufficiency with eliminated TAVI valve function	88/41 mmHg

The study confirmed the clinical results obtained by Dr. Hufnagel (1, 2). The difference was that he used classical thoracic surgery whereas we used catheter-based techniques. The study protocol was very extensive and physiologically challenging for the animals. Most pigs died from complications before all measurements were finished. Out of 24 experiments with aortic insufficiency, only 6 completed the study protocol. The complications were fatal bleeding from the extensive surgical procedures (*n* = 8), malignant arrhythmia (*n* = 6), thrombosis of the implanted TAVI valve (*n* = 3) and malignant hyperthermia (*n* = 1). These results have never been published before because we believed that the model introduced too much selection bias given the high mortality rates and variety of failure modes. But for historical information and documentation, the data are now revealed.

## *In-vitro* Experiments

**Figure 8:**
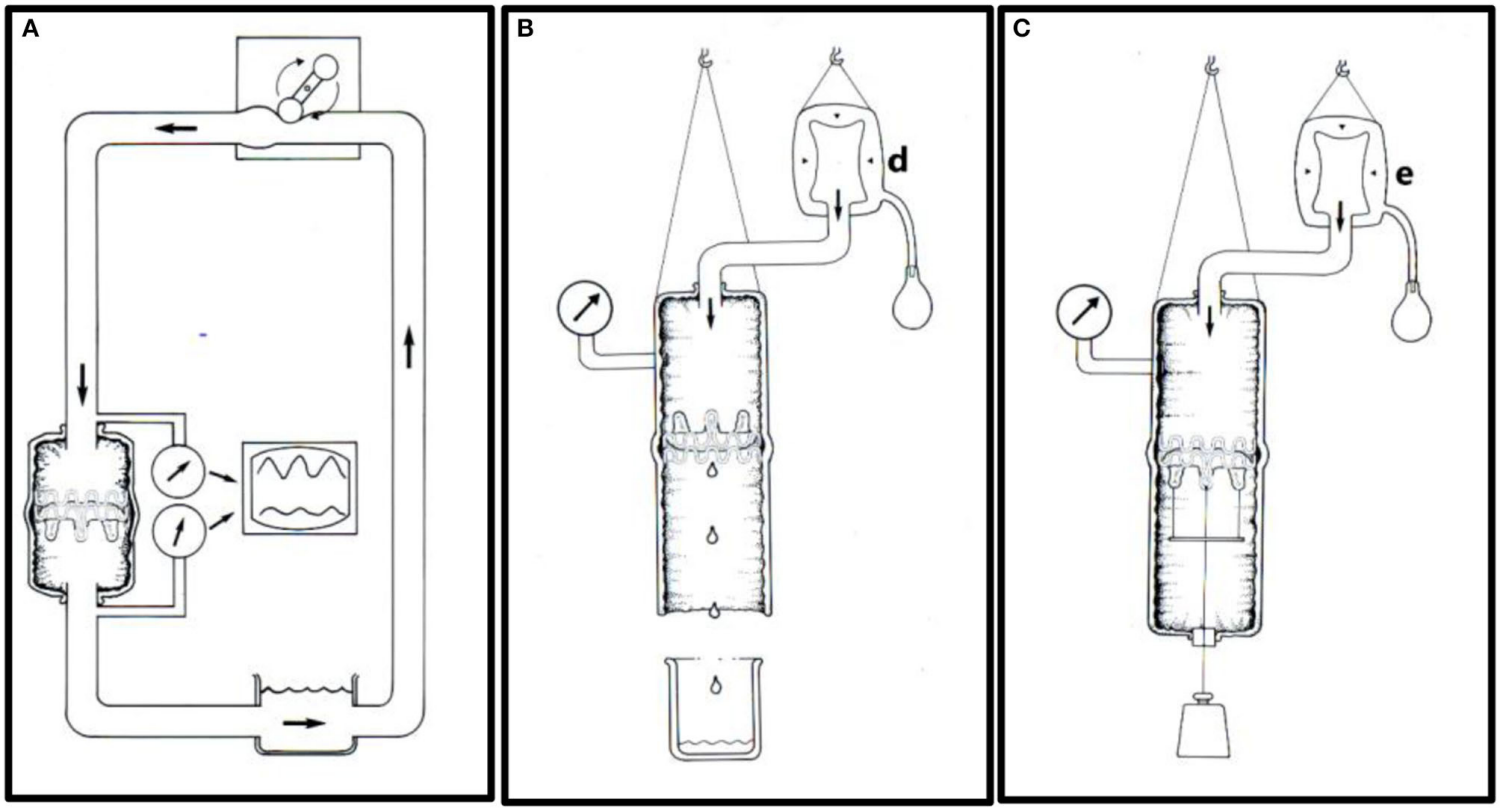
*In-vitro* testing. **(A)** An aortic specimen with an implanted TAVI valve mounted in an open circulation driven by a roller pump. Pressures were measured on each side of the TAVI valve to determine the pressure drop. The flow's direction is indicated by thick arrows. **(B)** Experimental set-up for leakage flow measurements. A constant downstream saline pressure of 100 mmHg was maintained above the closed TAVI valve by a clinical blood pressure infusion bag (d). The retrograde saline volume was measured simulating a combination of paravalvular and transvalvular leak. **(C)** Experimental set-up for measuring prosthesis stability. The TAVI valve was implanted in a porcine thoracic aorta specimen and attached to a metallic disc and connected to an external axial load of increasing weight from 0.5 to 2.0 kg through a silicon occluder. The experiments started at 0.5 kg and were successively increased by 0.5 kg every 3 h. A constant intraluminal pressure of 100 mmHg was maintained by a pressure infusion bag (e).

At the same time, simple *in-vitro* testing was performed (Figure 8). A total of 36 valves were implanted in isolated pig aortas using 25 mm (*n* = 18) and 31 mm balloons (*n* = 18), respectively.

In six valves dilated with 25 mm balloons and in six valves dilated with 31 mm balloons, transvalvular pressure gradients were measured using saline circulation at different flowrates from 5 to 8 L/min (Figure 8A). None of the valves became dislodged at these flow rates. In another 12 valves, the retrograde leakage volume was measured (Figure 8B). For this study, the aortic specimens with the implanted valves were hung up in a vertical position. A saline pressure of 100 mmHg was maintained above the closed valve by a fluid reservoir connected to a clinical blood pressure bag (d). Retrograde leakage was documented by measuring the fluid volume that leaked retrograde. None of the 12 valves dislodged during these experiments despite a retrograde pressure difference of 100 mmHg. Finally, the last 12 valves were tested for mechanical prosthesis stability to simulate dislodgement (Figure 8C).

## Scientific Presentation

**Figure 9:**
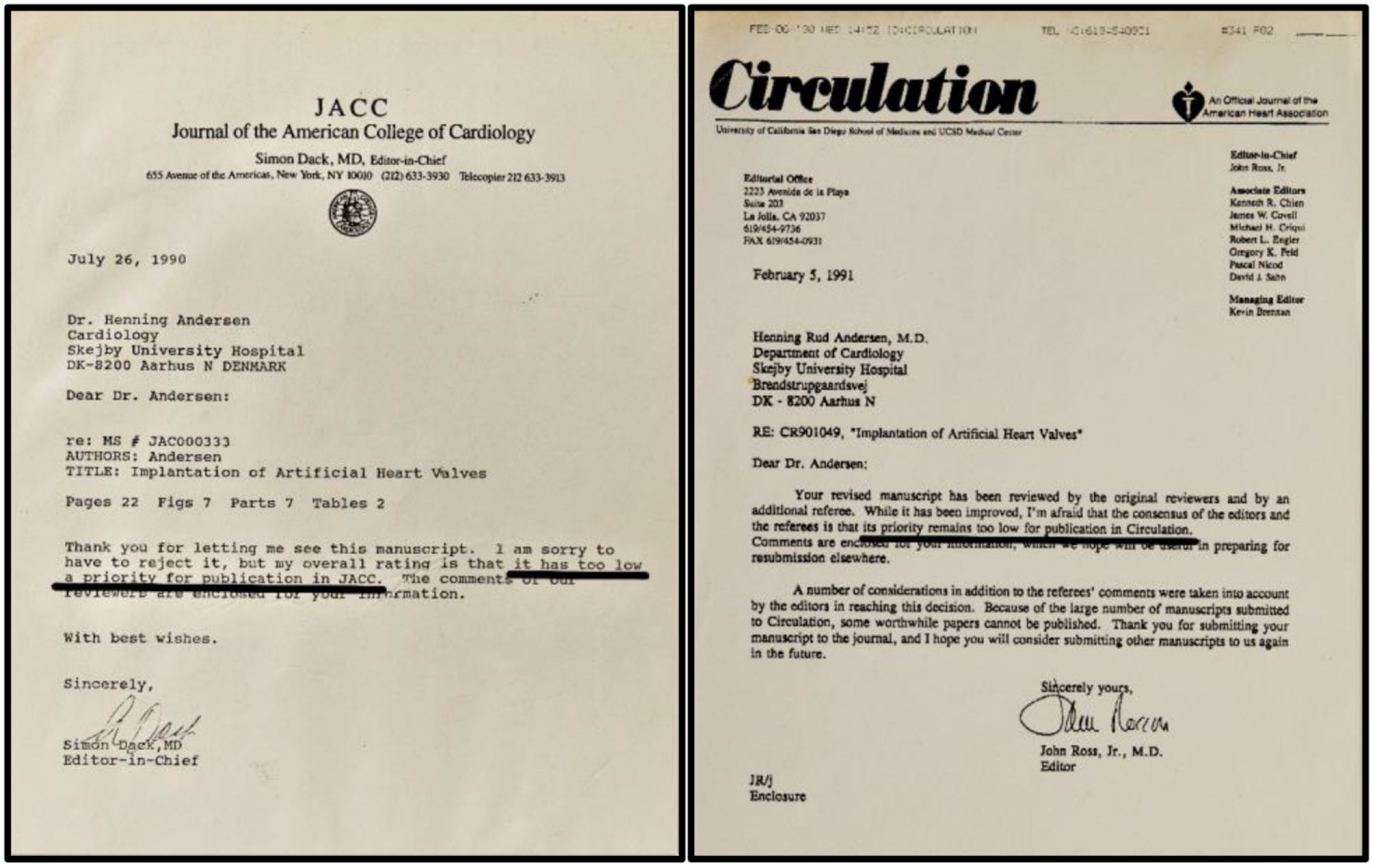
**Left:** Editorial letters from Journal of the American College of Cardiology, (1990). **Right:** Editorial letters from Circulation, (1991). Both journals declined publication with the argument that it had too low a priority for publication. The black underlining is made by the author.

The TAVI invention was presented for the first time on May 19, 1990, at the 30-year anniversary symposium of the Danish Society of Cardiology in Odense, Denmark. In May 1990, we had submitted another abstract to the 12th Congress of the European Society of Cardiology meeting in Stockholm, Sweden. The abstract was not accepted for presentation. In June 1990, we submitted a manuscript to the Journal of the American College of Cardiology (JACC) which at that time had an impact factor of 5.9. Two of the four JACC reviewers had many concerns such as “*lack of long term-term follow-up, lack of information about long-term durability, risk of dislodgment, risk of larger clot formation with peripheral and central embolization including clot embolization to the coronary arteries, risk of gradual dilation or even necrosis of the portion of the aorta where the valve is implanted with subsequent distal migration, lack of hemodynamic measurements with calculation of the Gorlin valve area.”* Therefore, the two reviewers did not recommend publication in JACC. Then the Editor-in-Chief reviewed the manuscript together with a cardiologist. The Editor did not reveal the name of the cardiologist but wrote to us that he/she was “*a cardiologist skilled in interventional procedures.”* The cardiologist responded quite differently from the two first reviewers. The Cardiologist wrote “*This is a most unusual manuscript, and the thinking is very creative and innovative. I think the manuscript is extremely well written and the data carefully collected and presented. Because the subject of the report is extremely controversial, at first there might be some reluctance to publish this work in some of the surgical journals. However, I think this would be a mistake and the data should be published in a respected journal. Because of the quality of the work and the possibility of long-term importance, I feel that the manuscript should be published. I think the Journal of the American College of Cardiology is an appropriate place.”* Finally, the Editor-in-Chief reviewed the manuscript himself and concluded “*the deficiencies are such that publication now is premature. The authors need to provide a longer follow-up after placement of this valve under pulsatile condition*.” Therefore, he wrote back to us in July 1990 “*I am sorry to have to reject it, but my overall rating is that it has too low a priority for publication in JACC”* (Figure 9, left).

The next manuscript was submitted to Circulation in fall 1990 after we had performed additional animal implantations. At that time Circulation had an impact factor of 9.0. One of the Circulation reviewers wrote “*I do not see any possible use of it in patients with calcified aortic stenosis*” and another reviewer claimed, “*the current report is very crude*” and “*aortic stenosis is not a place where this could be used*.” Indeed, in our manuscript we had suggested “*patients with calcified aortic stenosis who are treated with balloon aortic valvuloplasty might also benefit from implantation of the stent-valve*.” It seemed that our suggestion provoked both reviewers. The second reviewer continued “*questions regarding neointimalization, calcification, thrombogenicity, and dislodgement during long-term follow-up should be addressed*.” A third reviewer claimed, “*this paper is somewhat gimmicky*. *To simply state that you can do this on an acute basis is poor science and I think that it is giving the wrong message*.” Indeed, it was difficult for us to respond in a scientific way to these statements from the reviewers, so the manuscript was rejected, and the Editor wrote back to us in February 1991 “*its priority remains too low for publication in Circulation*” (Figure 9, right).

Finally, in March 1991 the manuscript was submitted to European Heart Journal, which at that time had a very low impact factor of only 1.6. The paper was accepted, and it was published in May 1992 (3). It was over 3 years after the idea sprang to life in Scottsdale.

**Figure 10:**
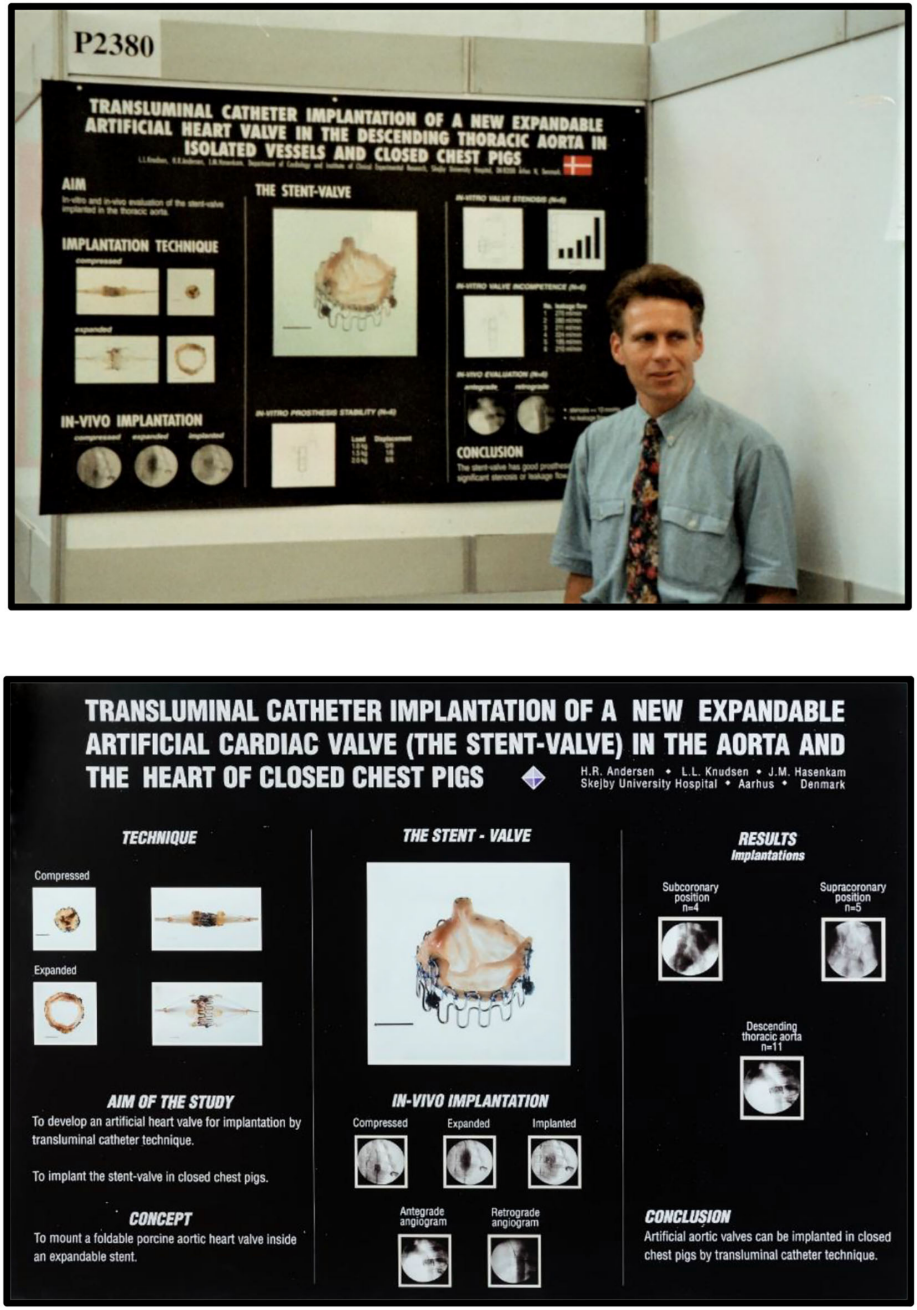
**Top:** Henning Rud Andersen with his poster at the 14th Congress of the European Society of Cardiology (ESC) scientific meeting, Barcelona, Spain, August 1992. **Bottom:** Poster from the 65th American Heart Association scientific meeting in New Orleans, USA, November 1992.

The next paper was published in 1993, also in a journal with an extremely low impact factor (4). An abstract was accepted for a poster presentation in 1992 at the 14th European Society of Cardiology Scientific meeting in Barcelona, Spain (Figure 10, top) (5).

Another abstract was accepted for poster presentation in 1992 at the 65th American Heart Association Scientific meeting in New Orleans (Figure 10, bottom) (6). Neither of the two publications nor the two posters attracted much attention. It seemed that the TAVI invention could not be published in major prestigious international journals with high impact factors or presented as oral presentations at international conferences.

## TAVI From 1989 and Beyond

Shortly after our first publication of balloon expandable valves in pigs, Professor Dusan Pavenik reported a percutaneous self-expandable mechanical valve which was successfully implanted in dogs (7). Pavenik's first-generation valve was a caged-ball design implanted in a two-step procedure. Later, Pavenik developed a mechanical disc valve which could be implanted in a one-step procedure (8). During the following years, several groups confirmed our concept using both balloon expandable and self-expandable biological valves in animal studies. Professor Philippe Bonhoeffer did preclinical evaluation with balloon implantation in the pulmonary artery in a lamb model (9), and in 2000 he performed the first-in-human percutaneous balloon implantation in a 12-year-old boy with stenosis and insufficiency of a prosthetic conduit from the right ventricle to the pulmonary artery (10). In 2001, Alain Cribier reported his experience with balloon implanted valves in sheep (11), and in 2002 he performed the FIM implantation in an adult patient with a severely calcified aortic stenosis (12). In 2004, Cribier reported implantation in six high risk inoperable patients using the antegrade atrial trans-septal approach (13). In the next series Cribier used both antegrade and retrograde approaches (14). These implantations were undertaken under mild sedation and in local anesthesia and without extracorporeal circulation. The studies were successful when we take into consideration that the patients were old, had multiple comorbidities, were in New York Heart Association (NYHA) functional class IV and several of them were in cardiogenic shock. All of them had been deemed inoperable and refused for surgery by two independent cardiac surgeons. It was said “*they had one foot in the grave and the other on a banana peel”* (15). A new self-expandable biological valve (CoreValve) was pioneered by the French cardiac surgeon Professor Jacques Seguin. Following initial implants in patients in India in 2002 (16), the first human implantation in Europe was performed by Professor Eberhard Grube in Germany in 2005 (17), followed by a registry study in 25 patients (18). These procedures were performed with the patient in general anesthesia and with percutaneous extracorporeal femoral-femoral bypass.

Initially, Cribier used the percutaneous femoral venous route, transseptal atrial puncture, balloon dilatation of the atrial septum, right atrium to left atrium access, mitral valve, left ventricle, and finally antegrade through the aortic valve where the 260-cm-long guidewire was advanced and snared from the femoral artery and externalized *via* the arterial sheath (12–14). The procedure was very complex and demanding and required extensive experience with cardiac catheterization and therefore complications were common. Consequently, the antegrade approach was largely abandoned with the advent of the transfemoral procedure in 2005 (19). Femoral, subclavian/transaxillary and transaortic access was developed (19–24). The first apical implantation in an animal was performed in 2000 by Professor John Webb in Vancouver, Canada (25, 26). Subsequently, the apical access was introduced in humans and soon became a preferred route for cardiac surgeons (27, 28). Recently, the carotid artery has been introduced as an alternative access route (29, 30). A few patients have also been treated with transcutaneous apical needle access through an intercostal space, and transcaval to abdominal aorta has been used in small series. In 2020 the first TAVI implantation in a porcine model through an interventricular septal approach was described (31).

Throughout the past 10 years, there have been tremendous development in valves, delivery systems, technical approaches and in experience of doctors to adapt the new technologies. New devices have been invented by creative doctors as well as industry who want to enter this new and very lucrative multi-billion-dollar market. It has led to many large randomized trials. The first such trial compared transfemoral TAVI with medical treatment including BAV in patients not suitable for surgery (32). TAVI significantly reduced the rates of death from any cause, but major strokes and major vascular events occurred more frequently in the TAVI group. After this first landmark study, a series of randomized trials comparing TAVI with cardiac surgery has been reported in patients at high risk (33, 34), intermediate risk (35, 36) and low risk (37–39) for cardiac surgery. The overall conclusion is that TAVI is superior or non-inferior to surgery, and 5-years follow-up confirmed these early results (40–43). Therefore, in patients ≥75 years of age, the totality of data demonstrated that TAVI should be the preferred treatment regardless of the degree of surgical risk (44).

Before TAVI was born, patients with severe aortic stenosis had only two treatment options, either surgical aortic valve replacement (SAVR) or medical treatment. This has changed significantly after the appearance of TAVI. Today, many more patients are referred for evaluation because TAVI might be an option for those who were previously declined for surgery. In many centers, including my own institution, first choice treatment for aortic stenosis is TAVI in 75–80% of patients, and SAVR in the remaining 20–25% of patients. The total number of patients treated for aortic stenosis has increased substantially since we can offer both SAVR and TAVI.

## The Heart Team Approach

The multidisciplinary TAVI heart team approach was established in the animal laboratory in 1989 with cooperation between interventional cardiologist, cardiac surgeons, anesthesiologist, doctors specialized in echocardiography and specially educated staff. This early collaboration between several specialties has proven to be a huge advantage for a very fruitful clinical collaboration. It has added tremendous benefit to the management of patients. In my institution it was natural for us to bring the heart team approach from the animal laboratory into the clinical setting. Therefore, many of the doctors who assisted me in the animal laboratory became members of our clinical TAVI heart team. Our first two clinical procedures were performed in 2006 with retrograde femoral artery technique and done in the cardiac catheterization laboratory. Unfortunately, both patients died during the procedure and our TAVI program was stopped. With a mortality rate of 100% for TAVI, we realized that we again had to learn from more experienced centers abroad. Therefore, we brought several of our doctors representing different specialties to USA and Canada to learn from experts. Additionally, we received on-site assistance from Professor John Webb, Vancouver, Canada when we successfully took-up the program again in 2007. Later, the TAVI team decided to move all TAVI procedures from the cardiac catheterization laboratory to the cardiac surgery department. A surgical suite was rebuilt into a huge hybrid room equipped with all modern facilities for TAVI. Today, our cardiac surgeons have been trained to perform femoral catheterization and valve implantations, and our cardiologist assists during the surgical TAVI procedures. Anesthesiologists are responsible for sedation, hemodynamic monitoring as well as the rapid- and back-up pacing. They also have the mandate to stop the procedure if low blood pressure or severe arrhythmias appear which need correction before the procedure can continue safely. The doctors and nurses from several specialties are now essentially transformed into hybrid doctors and hybrid nurses performing surgical and cardiology TAVI procedures together, thus eliminating the silos of the past.

## TAVI Circle Closed

**Figure 11:**
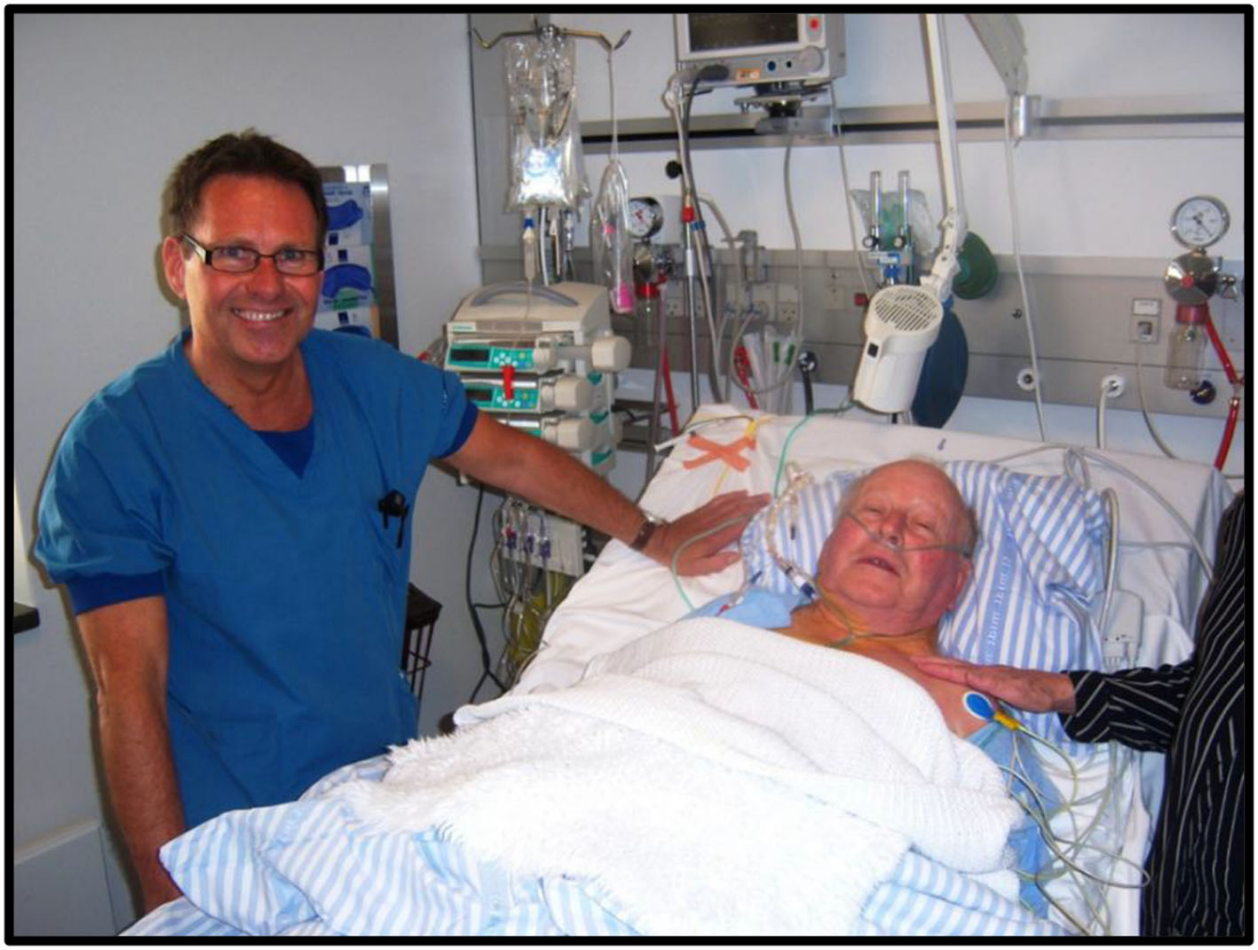
Henning Rud Andersen with his farther Jørgen Rud Andersen a few hours after percutaneous transfemoral TAVI in 2011.

In 2011, my 86-year-old father who was suffering from severe, symptomatic aortic stenosis was treated with a percutaneous transfemoral TAVI procedure (Figure 11). It was an enormous success for him. He was up walking around on the day of the procedure and discharged a few days later. He regained a normal life without any cardiac symptoms until he died 8 years later at age 95. For me, the circle was completed. My invention became a great personal achievement and the greatest gift I could ever give to my father.

## The Business World: The Andersen Patent

In 1989 we received assistance from the Danish Technology Institute (DTI) to draft a Danish patent application. DTI is an independent non-profit government institution with the goal to bring Danish inventions into production in Danish companies. DTI sought independent counseling from a Danish professor and interventional cardiologist. Based on his evaluation, DTI decided to grant sponsorship for a Danish and international patent application. The application covered the overriding fundamentals around a collapsible and expandable heart valve which included both balloon expandable as well as self-expandable valves. It described heart valve implantation in the aortic, pulmonic, mitral, and tricuspid positions. But the patent was not confined to heart valves. The title was “*Valve Prosthesis for implantation in the body and a catheter for implanting such valve prosthesis”* (Figure 3). Because it was so disruptive, it also included percutaneous catheter implantation of artificial valves in all places of the body where fluid is transported. The idea landed on virgin ground, and therefore it was easy to obtain a worldwide patent. It was so strong that it proved impossible to circumvent. It was later—unsuccessfully—challenged by attacks from large companies in patent courts. They tried to claim it was invalid and that it should be revoked because they wanted to enter the lucrative multi-billion market which was protected by the patent. However, the patent prevailed and survived in all patent fights in Europe and USA. It soon became known as “the Andersen patent” in the industry and in the world of patent lawyers.

With the Danish patent application in hand, we approached several Danish medical device companies. All the companies told us it was very interesting, but they declined to enter the project. They found that the development challenges and the costs would be too high. It would be a too risky investment and stretch over at least 10 years before a positive business might, or might not, materialize. We subsequently approached several other European companies with the same discouraging outcome. At that stage, DTI concluded that we would not be successful in finding a company in Europe, and therefore DTI could no longer sponsor our efforts to promote the invention with resources granted from the Danish government. We were left to try ourselves without further assistance and support from DTI.

In 1992, after 41 *in-vivo* experiments, we realized that we had to find a non-European company with expertise in heart valves to develop the invention. We could no longer achieve more by building non-sterile valves and catheters with our own hands and performing only non-sterile acute feasibility studies. We needed serious sponsorship to continue the animal experiments with sterile heart valves and long-term follow-up studies.

With the international patent and our scientific results in hand, I contacted many of the big players on the market at that time; Johnson & Johnson (J&J), Medtronic, Baxter B.V. which at that time owned Edwards Lifesciences (Edwards), Boston Scientific, St. Jude Medical, USCI-Bard, SCIMED, Trimedyne, Meadox Surgimed, Pfizer, Astra Meditec and more. My efforts were all in vain. The response from the companies was always the same: “*Wow, that's interesting, I've never heard of a percutaneous heart valve before, We will look into it.”* And who did the companies call to seek advice? Who were the experts in heart valves? The cardiac surgeons, of course! And what did the cardiac surgeons say? “*It is a silly idea, with very few patients who need it, after all there is no such thing as a nonsurgical patient. We can operate all the patients and have perfect outcomes. Here are the top eight reasons why this is never, ever going to work. It's a ‘ridiculous’ idea,”* (repeated eight times, authors comment). It was said that cardiologists know nothing about aortic stenosis and should not treat these patients, and “*It is the most stupid project ever heard of.….It will never work”* (15).

## Licensing the Patent to Stanford Surgical Technologies

**Figure 12:**
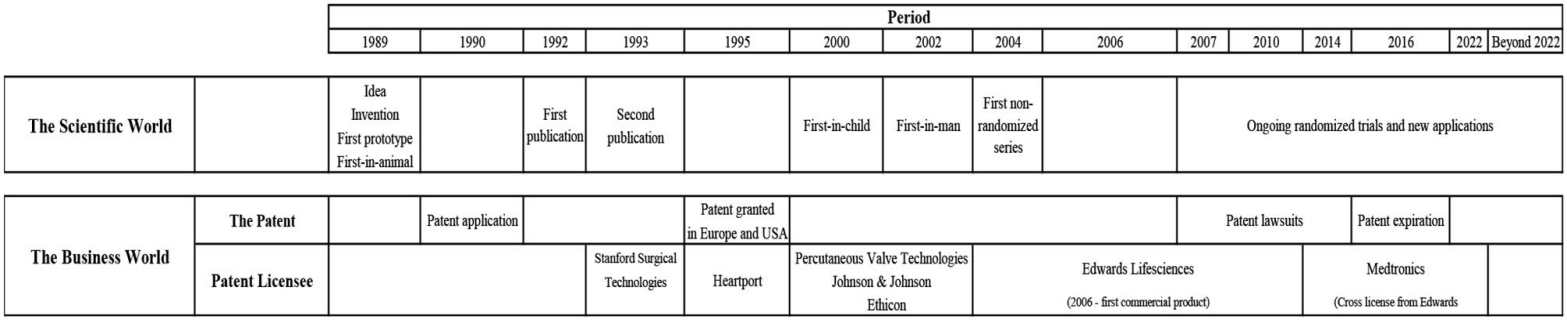
Timeline 1989 – beyond 2022.

In 1993, I could no longer afford to pay the yearly costs to maintain the patents. I had to find a sponsor to pay for maintaining the patents or lose them. A small company, Stanford Surgical Technologies (SST), from California had contacted me in 1992 during my poster presentation at the 65th American Heart Association Meeting (Figure 10). SST wanted to purchase the patent, but I would not sell it. Instead, in 1993 I decided to license it to SST in exchange for their payment of the patent fees which could keep the patent alive (Figure 12). We still owned the patent so we believed we could control the fate of it. It was a strategy I had learned from listening to Julio Palmaz's lecture in 1989. Palmaz licensed the rights to his patent to J&J, but he maintained ownership of it, a strategy which paid off for him.

During my visit to SST in California the same year we discussed how we could move forward together. My ultimate goal was to take my invention to much higher levels and finally develop it into a clinical treatment for patients, and at that time I judged that SST was capable of taking the first steps together with me. After returning to Denmark, SST wrote to me “*Our first goal is to send you sterilized valves for the 1–2-month chronic studies we discussed. I will keep you updated on the progress of this development.”* I never received one single valve or catheter from SST for the years to come despite several inquiries which gradually became aggressive.

Due to our financial constraints, and our inexperience in the world of business and contract law, our position for negotiations of the license agreement with SST in 1993 was very weak. Therefore, we ended up with a very flawed agreement. We could not afford to hire our own independent lawyers to assist us with negotiations. The patent applications nearly expired during my negotiations with SST. They delayed the writing and signing of the agreement until the very deadline for the next payment of the patent renewal applications and we nearly lost the patent. I had to raise private bank loans with a mortgage in my own home to ensure the survival of patent for 12 more months until the license agreement was finally formulated and signed, a situation which SST took great advantage of. Our very weak license agreement granted SST a worldwide exclusive license, including the right to grant sublicenses, and sell the license agreement to other companies without our engagement and without the obligation to share any income derived from that. The agreement did not include anything about the inventors' rights to be involved in the company and nothing about our participation in development, scientific research or academic publications. Furthermore, the license agreement did not include a clause about SST's obligation to develop the invention within a specific time frame. They were even free to do nothing, which turned out to be the case. We received an initial upfront payment of $20,000 to share between the three of us, and 5 years later we began to receive a fixed amount of $10,000 every year. The agreement also comprised a clause of 2.5% royalty from sales, but we never reached the point where we received royalty. It was very different from the decent deal Julio Palmaz negotiated with J&J when he licensed his stent patent in 1988. Palmaz received an initial down payment of $10 million plus royalty for 10 years which according to Shawn (45) amounted to “about $500 million” when Palmaz sold his patent to J&J in 1998.

## Transfer of the License From Stanford Surgical Technologies to Heartport

SST was founded by cardiac surgeons from Stanford University Hospital. They promised me to develop the technology and pay the fees for maintaining the patents. But SST did not reveal that they had already invented a new surgical technique, the minimally invasive Port Access Surgical Method for less invasive surgical aortic valve replacement. It turned out that SST wanted to develop their own invention rather than my device. SST soon changed their name to Heartport to reflect their own invention, Heart-Port-Access, and transferred the TAVI license agreement to the new company (Figure 12). Two years later Heartport announced they had also developed a new port-access technology for Coronary Artery Bypass Grafting (CABG) surgery. Now they owned a technology to perform both Port-Access-Valve Replacement combined with Port-Access-CABG. It became obvious to me why they did not develop the TAVI invention which was a potential competitor to their own invention and patents. In1995, Heartport and St. Jude Medical announced they had “*entered into a worldwide agreement including provisions for product development, patent licensing, and component supply, as well as the sale by St. Jude Medical of a new, jointly-developed product, the “St. Jude Medical Port-Access Mechanical Heart Valve System, incorporating Heartport Port-Access Technology.””* Now it was even more evident why TAVI was never developed in SST/Heartport and why they wanted to pay for the patent to make sure that other companies would not develop it. The license agreement survived deep in their archives. I complained aggressively to the CEO of SST/Heartport several times, but to no avail. He never responded. Heartport did pay the yearly patent fees which kept the patent alive, but my idea languished for 8 years and no development work was done despite their promise. In 2001, Heartport wrote to me, “*Heartport, Inc. had not pursued the development of licensed product based on the Licensed Patents. I like to hereby inform you that we signed a sublicense agreement with Percutaneous Valve Technologies in December 2000. The sublicense is in full compliance with the original Agreement. Enclosed please find a copy of the sublicense agreement. Also enclosed, please find two press releases.”* So now, the license agreement had moved into a new company and the financial deal between the two companies had bypassed us.

## Transfer of the License From Heartport to Percutaneous Valve Technologies and Johnson and Johnson

Three weeks later Heartport was purchased by J&J for ~$70 million, but at that time Heartport had already sold the cardiovascular part of the license agreement to Percutaneous Valve Technologies (PVT) for up-front $1.0 million followed by $2.0 million 2 years later, and 3.5% equity in PVT. The rest of the license agreement was acquired by Ethicon, Inc., a J&J subsidiary, as was Heartport itself (Figure 12). Consequently, J&J had bought 3.5% equity in PVT, and therefore had a business interest in our TAVI license agreement. Paradoxically, it was J&J that had purchased the exclusive rights to file and prosecute new cardiovascular patent extension applications, not PVT. It led to much confusion when we found out that several new patent applications were filed in the name of us three patent owners but with Heartport wrongly assigned to the patent and without our knowledge. It also took me several communications with the different companies to find out who should now pay the yearly patents fees to ensure survival of our patents. It turned out to be Ethicon and not PVT or J&J.

When I realized that Heartport did not develop my invention, I contacted J&J and asked them if they were interested. At that time, they had already filed one of Alain Cribier's patent applications about TAVI valves, but so far, they did not develop it. J&J's Vice President of New Business Development called me and asked questions about our patent and the license. He was interested to discuss licensing the patent. I met with him and his co-Vice President in California together with a group of key persons from J&J. I wanted J&J to unbind the license from Heartport based on the fact that they did not develop it. My plan was to offer the license to J&J if they were seriously interested in developing it. Since they had already filed one of Cribiers patent applications I hoped they would say yes. The legal judgment from the J&J group was that it was not possible to unbind the license according to California laws. Then I suggested that J&J simply buy the whole company to get control of the license. The people from J&J liked the idea and went to the top management of the company and recommended it, but top management was not interested.

## Foundation of Percutaneous Valve Technologies

The two Vice Presidents from J&J became increasingly interested. They left J&J and began negotiations with Heartport to purchase our license agreement. They realized that if they were going to build a new successful company, they had to own the license to the Andersen patent. PVT was established by four people, the two previous Vice Presidents from J&J, Professor Marty Leon from New York and Professor Alain Cribier from Rouen in France. PVT raised ~$19 million in funding capital to acquire the license and to develop the idea. Several U.S. companies such as Medtronic, Boston Scientific Corp., J&J and Oxford Bioscience together with two Israelian companies, Aran Research & Development and Medica Venture Partners invested in PVT and they all became members of the board of the company. I established a non-commercial partnership with PVT, but I had no money to invest. Much of the initial development was performed by Aran Research and Development Ltd. in Israel. Animal implantations were performed in Paris. The FIM implantation was performed by Professor Alain Cribier in Rouen, France in 2002 and was a great success (12).

## Transfer of the License From Percutaneous Valve Technologies to Edwards Lifesciences

In 2004, PVT and the license agreement were purchased by Edwards for $125 million in down payment and up to an additional $30 million upon achievement of key milestones (Figure 12). It was a difficult decision for Edwards to acquire PVT. Edwards was the world's leading manufacturer of surgical heart valves and their customers were cardiac surgeons. Several members of their advisory board were also cardiac surgeons. They were against it because TAVI was not about cardiac surgery but interventional cardiology and they did not recommend Edwards to engage in such a radical shift in business strategy. When Alain Cribier had performed the FIM and small series of implantation with the PVT valve (12–14), Edwards' CEO realized that his company was far behind with their own in-house TAVI development, and PVT was several years ahead of them. The Edwards' CEO acknowledged that PVT could undoubtedly sell their company for a considerable sum to one of Edwards' competitors which would outperform them and leave Edwards as a minor company in the heart valve market. The PVT's CEO deeply favored Edwards to develop their technology because Edwards was the leading heart valve company in the world. They had all the expertise needed, but he also realized that Medtronics, Boston Scientific and J&J, which were already members of the PVT board, had much stronger finances and could buy PVT for a huge amount and thereby outbid Edwards. Then, a very elegant strategy was orchestrated by the PVT's CEO. First, he called the Edwards' CEO and arranged a confidential meeting. The PVT's CEO suggested a strategy which could resolve the problem with overbidding from the much wealthier companies. The two CEOs agreed that Edwards make a bid of $125 million in cash without any term sheet. Both of them predicted that the other companies could not match such an offer in cash in such a short notice. It was all about timing and cash. After thorough planning, the PVT's CEO unexpectedly gave the other companies only 72 h to decide if they would give a higher bid in cash or leave it. He showed them the contract which Edwards had already signed. The other companies could predictably not make such a decision within 72 h, especially not in cash and without the opportunity to negotiate a term sheet agreement. Obviously the contract went to Edwards [Figure 12, (15)].

## Further Development of TAVI In Edwards Lifesciences

**Figure 13:**
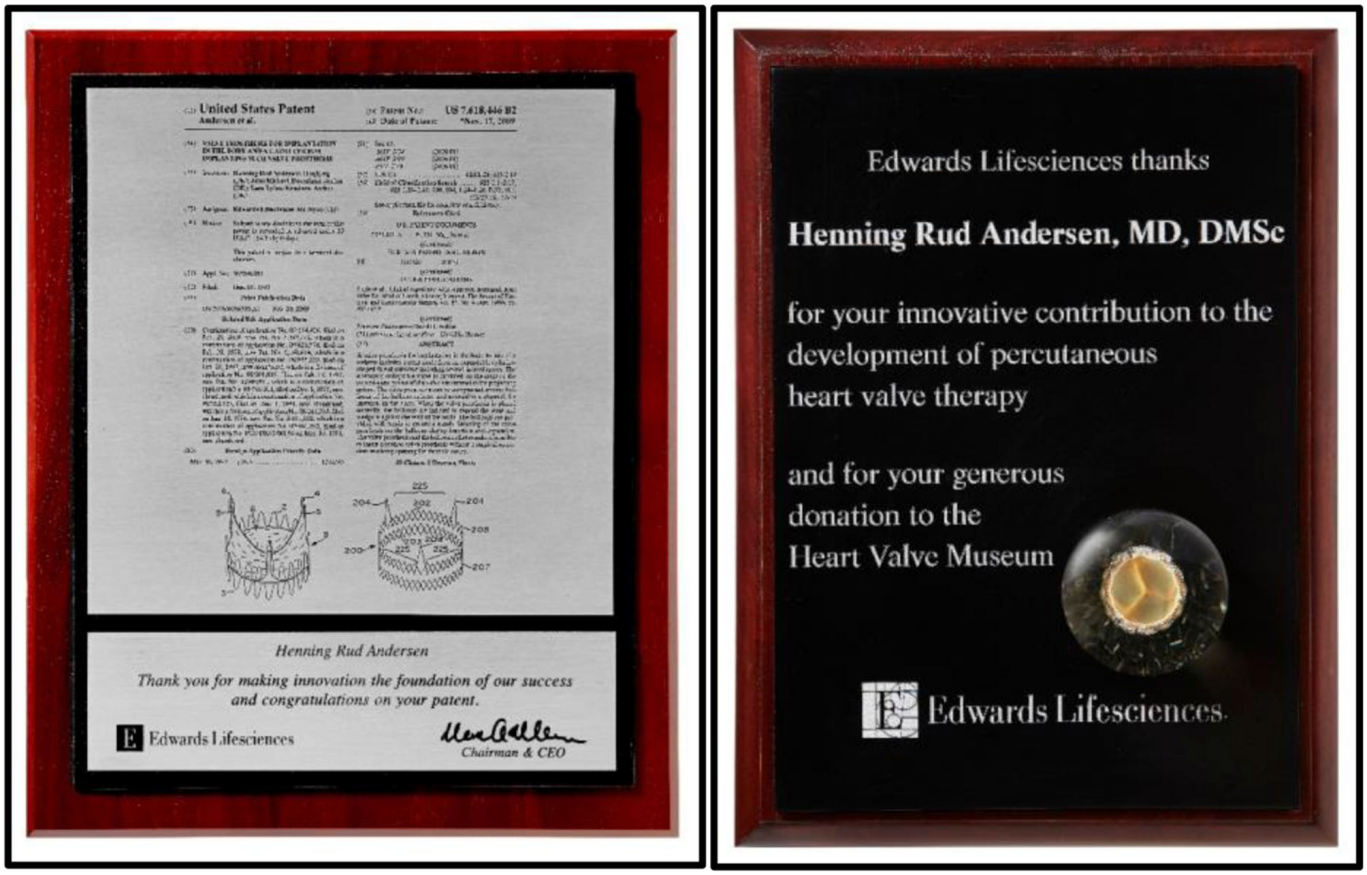
**Left:** Acknowledgments from Edward for innovation of TAVI and foundation of Edwards' success. **Right:** Acknowledgments from Edwards for development of TAVI and for donating TAVI prototypes to the Edwards Heart Valve Museum in Irvine, California, USA.

PVT now moved from their headquarter in New Jersey to the Edwards headquarters in Irvine, California together with key persons from PVT who all became integrated into Edwards. The PVT team established their own research and development unit inside the Edwards company where they got unrestricted hands to develop TAVI. Much to my disappointment Edwards decided to name the valve “the Cribier-Edwards valve.” That choice of name for the valve infuriated me because PVT and Edwards had on several occasions acknowledged that the TAVI valve was based on my invention and the Andersen patent (Figure 13).

After tough face-to-face meetings with Edwards where they argued that Cribier was very famous and I was not, which was actually correct, they finally accepted my point, and the name was soon changed to the more neutral Edwards SAPIEN valve. Edwards continued to invest, develop and refine the TAVI technology and made it into a lifesaving treatment for thousands of patients. It also became an enormous financial success for the company. I continued to be a partner in the non-commercial success story with Edwards. The original patent was still owned by us, but we got nothing from the deal between PVT and Edwards and no royalty from Edwards' annual sales of the SAPIEN valve which in 2021 alone is estimated to be $3.5 billion.

## The Fight for the Patents: Corevalve V. Edwards Lifesciences

In 2007, a patent fight started between the French company CoreValve Inc. (CoreValve) and the Andersen patent. CoreValve had developed a self-expandable TAVI valve which infringed the Andersen patent because we owned the intellectual rights to the design. The patent fight initiated a legal battle with multiple court confrontations in Europe and USA for the next 7 years. First, CoreValve claimed that the Andersen patent was invalid and should be revoked. They filed a patent lawsuit against us patent owners in London. In 2005, I had been contacted by CoreValve, who at that time was located in Paris. They invited me to enter into a consulting agreement with them and they promised to compensate me amply for my services. But during this period I already had a partnership with Edwards and did animal implantations in Irvine, California, educational lectures in the company and scientific lectures at Edwards symposiums in Europe and USA. Therefore, I felt that I could not work with two competing companies. CoreValve did not inform me that they planned to file a patent case against us three patent owners in London. We first found out when we received a strict confidential personal letter from The High Court of Justice in London in 2007 where we were required to show up in London to defend our patent. I was in disbelief that, I was offered a consultancy position with CoreValve and shortly thereafter to be the target of a patent case. Shortly before the court hearings began in London, Edwards suggested that they could defend the patent with their lawyers free of charge for us three patent owners. We did not have our own lawyers and we could not afford to hire legal representation. Fortunately, we did not immediately accept Edwards wish to acquire ownership of the Andersen patents. At that time, Edwards had already invested a huge amount of money in development and clinical trials and had obtained approval for commercial sales in Europe in 2006 (Figure 12), so the patents was of extremely high value for them. Therefore, it was very much in their interest that the patents survived in litigation because they did not want to share the European and USA market with other companies.

At the same time, Edwards had filed litigation against CoreValve in Düsseldorf, Germany claiming that the CoreValve patent infringed upon the Andersen patents. CoreValve had moved from Paris to Irvine in California and the fight continued in the USA. In 2008, Edwards filed new patent infringement litigation against CoreValve in USA. The suit sought injunctive relief and damages for infringement of the Andersen family of patents.

## The Fight for the Patents: Medtronic V. Edwards Lifesciences

In 2009, Medtronic purchased CoreValve for $700 million plus two $75 million milestone payments and continued the patent fight in USA even more aggressively (Figure 12). In 2010, a U.S. Federal jury ruled that the Andersen patent was valid, and that Medtronic willfully infringed it. The jury awarded Edwards $72 million in damages and $1.3 million as a reasonable royalty, and the willfulness finding allowed Edwards to seek increased damages of up to three times that amount (46). Medtronic appealed the ruling but in 2012 the U.S. Court of Appeals affirmed the decision. The Appeals Court also spontaneously ordered the Federal Court to reconsider Edwards' request for a preliminary injunction which would prohibit the manufacture and sale of the Medtronic's ReValving system in USA because the judge found “*There is evidence that Medtronics may have sought to stockpile infringing devices in the United States after the verdict* (2010 court ruling, authors comment) *as part of a greater plan to overtake Edwards in the THV market”* (46).

Several court meetings were held in USA, where J. Michael Hasenkam represented us three patent holders and appeared in the court in Wilmington, Delaware to support Edwards. In 2014 the Federal Court granted the injunction which required Medtronic to stop sales in USA within 7 days. The Court also ordered, “*The parties shall immediately enter upon discussions to jointly determine a mechanism by which a sufficient number of CoreValve Generation 3 devices can be provided to the hospitals and clinics that are currently already trained in use of the CoreValve Generation 3.”* The Court ordered that within 36 days, “*the parties shall apprise the court via teleconference of the status of their discussions.”* The Court did not expect that the discussions would end up with an overall settlement within such a short time, but the Court wanted to make sure that negotiations between the two companies had been initiated.

## A Patent Cross-License Agreement

On day 35, Edwards and Medtronic announced they had finalized a complete agreement to dismiss all pending cases worldwide in the field of both transcatheter and surgical valves for the 8 year duration of their agreement. Under the terms of a patent cross-license agreement, Edwards had granted Medtronic the right to manufacture and sell the Medtronic ReValving system under the Edwards' license (Figure 12). Medtronic made a one-time payment to Edwards of $750 million in cash. Additionally, Medtronic pays Edwards an ongoing royalty through April 2022. Royalty payments are based on a percentage of Medtronic's CoreValve sales, subject to a minimum annual payment of $40 million. So now, both Edwards and Medtronic benefited from their multi-billion-dollar business based on my invention and they had established a deal where Medtronic continues to pay royalty to Edwards until several years after the expiration of the Andersen patent. I was disappointed, because after all it was my invention and intellectual property the two companies were trading about. I had anticipated that they would be fair and reach out to us and rectify our flawed license agreement so the settlement also rewarded the innovators, but they did not. This is an important lesson for new inventors to learn from.

## Survival of the Andersen Patents

When the litigations began in 2007 nobody knew that TAVI would be such an enormous financial success. But we knew that financial success for Edwards and us, if any, depended on the value of my invention and the survival of the Andersen patent. Therefore, Edwards and we three patent owners formed a partnership to make sure that the Andersen patent prevailed in all litigations. If we turned out to be successful together with Edwards, we three patent owners should have a fair and substantial share of the outcome, if any, as long as our mutual partnership existed. Furthermore, we promised Edwards to participate in meetings with United States Patent and Trademark Office (USPTO) and help Edwards to ensure extension of the survival period of the Andersen patents beyond the timetabled expiration date. Our meetings with USPTO were successful and resulted in prolongation of the survival time of the patents by 2½ years into 2016. In the patent litigations we worked hard together with Edwards for 7 years in countless meetings in Europe and USA, and in 2014 the Andersen patents had finally prevailed and survived in all litigations in all countries. So far, both parties had fulfilled the obligations of our partnership. Edwards was granted a huge financial compensation of more than $1 billion. However, unexpectedly Edwards' compensation suddenly bypassed us and was never shared with us as they had promised. For new inventors, such a maneuver is important to remember and learn from.

After the 2012 ruling, Edwards had prevailed over Medtronic in all previous litigations. All USA judges had in unanimously declared that Medtronic willfully infringed the Andersen patents. In addition, the judges in several USA Courts were infuriated with Medtronic when they found out that the company gave misleading evidence and a judge claimed that “*Medtronic was well aware that its representation in courts was false when made”* (46). Furthermore, the Supreme Court denied hearing Medtronic's appeal. This was the situation before the final 2014 court ruling. With such a legal position, the likelihood that Edwards would prevail, and Medtronic would lose the final court ruling must have been obvious to both companies. Furthermore, they were exhausted after 7 years with endless and costly patents fights in Europe and USA. Therefore, we speculate that the scene was set for an early voluntary settlement, and today we believe that such settlement negotiations about the Andersen patents were already secretly initiated long before the final 2014 ruling.

In 2014 we met with Edwards' Head of Legal affairs in Denmark a few weeks before the settlement with Medtronic unexpectedly for us went public. The lawyer told us that the litigation with Medtronic was still very much alive and that it was likely to drag out for years, and that the outcome was completely unpredictable. She said that if we and Edwards lost the ongoing litigation, we three innovators would not receive anything in accordance with our partnership agreement. When we questioned the lawyer, she said she had additional information, which she could not reveal to us due to US disclosure rules. Today we suspect that she knew very well that the settlement between Edwards and Medtronic was already completed and merely awaiting publication while negotiations with us could be consummated so Edwards could save a huge amount which was due to us according to our partnership agreement. She then suggested that we end our 7-years-long mutual partnership, so we three patent holders at least got some compensation from Edwards instead of waiting maybe several years and risk losing everything. We were against it because we had fought hard together with Edwards for 7 years and so far, we had prevailed in all similar litigations against CoreValve and Medtronic in both Europe and USA. Therefore, we predicted we would prevail again in the final litigation which could reward us with a huge share of Edwards' compensation. The lawyer telephoned the Edwards' Headquarters in USA several times during the meeting and then she started to threaten us with year-long and costly litigation in USA courts if we did not accept their suggestion. I had never imagined that I, the inventor of TAVI, would be threatened with litigation from the world's leading heart valve company, particularly after having worked so hard together for so many years to defend the Andersen patents and to bring the invention to clinical and financial fruition. However, the increasing legal pressure on us from the lawyer and the Edwards' Headquarters in California became so strong that we gave in to it. It ended our partnership with Edwards prematurely. To our amazement and consternation, a few weeks later, and not “years” later in contrast to what was explained to us by the lawyer, the settlement between Edwards and Medtronic suddenly went public. We then realized that we had been maneuvered out of a fair and substantial share of the $1 billion compensation to Edwards few weeks before the settlement was announced. We had lost both our partnership and fair compensation and Edwards had saved an enormous amount of money. Today, we believe that the Edwards' lawyer knew very well that the settlement with Medtronic was already finished when she traveled to Denmark to end our partnership prematurely. Such strategies from companies toward inventors is worth remembering for new inventors.

At the time when we entered the partnership with Edwards, we were not aware that we, the patent owners, had the right to settle the dispute between us and the counterpart (CoreValve and Medtronic) because it is normally the patent owner, not the licensee, who can negotiate a settlement with the counterpart in patent fights. Indeed, Edwards would not like it because they had invested a lot of money, but we could probably have negotiated a much better deal with both Medtronic and Edwards if we had offered Medtronic a cross-license settlement agreement where they would compensate us with a substantial amount. Because both companies were very much interested in the patent and the huge business opportunity the patent controlled, we could have negotiated a license agreement with both companies and received a lifelong double royalty income from both. But Edwards' lawyers did not inform us about our legal rights to settle the dispute with Medtronic, and we did not have our own independent lawyers to advise us. Instead, Edwards single-handedly negotiated a huge settlement with Medtronic and sold a cross-license to them which bypassed us. This is also worth remembering for new inventors. Keep ownership of your patent so you can control the fate of it and hire your own lawyers. This was the background for Julio Palmaz's success when he finally sold his patent to J&J in 1998 for a huge amount (45).

## A Doctor in Patent Courts

Instead of fame and fortune, I did however, get an incredibly unique experience in life from the invention of TAVI, which I would never have obtained in any other way. It was the life in patent courts in several countries around the world, all crowded with numerous lawyers and judges, many of them dressed in black medieval gowns and long white wigs, fighting and judging about my intellectual property. A deposition was also held here in Denmark where Medtronic/CoreValve sent a group of their lawyers to question the three Danish patent holders in a hotel conference room which was officially converted into a USA court room for the deposition. We were supported by a group of Edwards lawyers sitting on the opposite side of the table. All three of us were individually grilled for several hours and everything was video recorded during the hearings.

**Figure 14:**
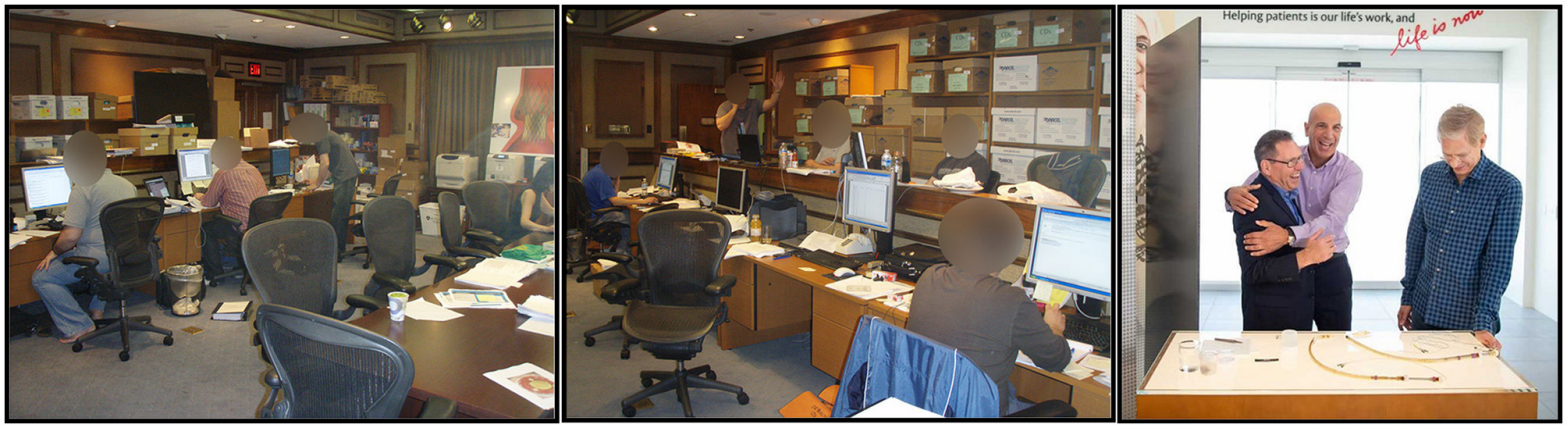
**Left and Middle**: “The War Room” for the patent fights, where all Edwards' legal preparations, meetings and storage of documentation were placed in one big, fully packed room in Wilmington, Delaware, USA in 2010. **Right**: After Edwards had prevailed in all patent cases, Edwards' CEO (left in the photo) was exited and gave me a warm hug. To the right in the photo is the CEO of PVT.

Afterwards, our partnership with Edwards was to participate in court hearings worldwide and in meetings with their lawyers and to hand over all relevant information and all raw data including early prototypes of the valves. The workspace for Edwards's law team—called “The War Room”—was a beehive of people, computers and documents set up in a hotel next to the court building in Wilmington, Delaware, USA (Figure 14).

In the Court room, the Medtronic lawyers twisted the wordings and claimed my idea, studies and publications were useless and therefore the Andersen patent was invalid. The Edwards lawyers on the other hand claimed the data, publications and patent were valid and that the Medtronic/CoreValve design was included in the Andersen patent because it clearly described both balloon expandable as well as self-expandable valves. Consequently, Edwards claimed that CoreValve infringed the Andersen patents and the courts agreed. After 7 years with court meetings the patent finally prevailed in 2014 which shortly after resulted in the cross-license agreement between Edwards and Medtronic which is still in force until 2022 (Figure 12).

## Discussion

In 1989 in Scottsdale, I had learned from listening to Julio Palmaz that an inventor must never ever give up. He must fight for his idea and his intellectual property, also if everything looks futile. That turned out to be sound advice because when an inventor comes up with a “crazy” disruptive idea, he must expect to be met with resistance and skepticism, even from his own peers and academic societies. A very disruptive invention like TAVI creates a paradigm shift and changes the world's concept of normality, and it creates resistance and annoyance when the inventor claims that the established rules no longer define the game (47). When the inventor announces that cardiac surgery is no longer needed to implant artificial heart valves many colleagues will not believe him, and some of them will even respond with anger. This response is widespread, even to the level of editors and review committees of scientific journals. In my case, disbelief and widespread rejection lasted 17 years before the first commercial valve reached market. This illustrates how much you must struggle and fight for your idea and during that time the clock is ticking on your patent.

## Advice for Inventors

It is important for you to file a patent application before contacting a company. Otherwise, you have nothing to offer and nothing to negotiate with. The company can simply take your invention for free. Furthermore, the invention has a higher value for the company if you have already protected it with a patent application or a patent. You should insist that the company sign a confidentiality agreement. However, be aware that it might be of minor value for you. If the company breaks the confidentiality agreement, the only course of action is to pursue litigation that will be very time consuming and expensive. All the while, once again the patent clock is ticking. You should hire your own lawyers from the very beginning. If you cannot afford it, the company must agree to sponsor the costs of your independent representation. If they do not agree to that, you should not negotiate with them. Remember, they can be very cold-hearted and unscrupulous, and they do not want the inventor to be in a strong legal position during negotiations. Sad to say, but they want to take advantage of your relatively poor financial position, your inexperience in the world of business and contract law, and your desire to have your brainchild developed and ultimately deployed. You should also demand that the company invest and develop your invention within a specified time, otherwise the license should be returned to you free of charge so you can find another company, and you should not accept to compensate them for their expenses. It is their failure and problem if they are not successful in developing your idea. That is the risk they assume. You should insist on being compensated appropriately with initial down payment, milestones, royalties and equity in the company. If the company is a small start-up company like PVT, it is preferable to acquire equity than take money from the company since the start-up company needs the money for development of your invention. Equity will gain value if your invention turns out to be a financial success either in the company itself or from a bigger company which acquires it. That was the case with PVT when Edwards purchased them. Importantly, the license and the financial agreement should remain in effect lifelong, even after expiration of your patent. This can be illustrated with my own TAVI story. The time from patent application until commercial sales was long (Figure 12). Our Danish patent application was filed in 1990, the European application in 1991 and the USA application in 1993 and again in 1994. The European and the USA patents were granted in 1995. The first approval for commercial sales in Europe was granted to both Edwards and CoreValve in 2007 and for sales in USA to Edwards in 2011 and to Medtronic in 2014. Consequently, the time from first patent application until first commercial sales in Europe was 17 years, and in USA 21 years, which is the lifespan of a patent. It is quite normal that transition from a ruling paradigm like cardiac surgery for treatment of aortic stenosis to a new paradigm like TAVI can last 15–20 years or more (47). It means that your patent can expire before commercial sales begin and you get no royalty from sales if the agreement is patent-related and not lifelong. However, your idea, invention and intellectual property remains lifelong and therefore the royalty should indeed also be lifelong as long as the company earns an income from your invention. In our case, we never received royalty from sales. However, still today in 2021, royalties are paid between Medtronic and Edwards based on sales of my invention. It demonstrates, that in the world of big multinational companies, royalty can be granted between companies long after the patents have expired. Therefore, the same rules should also apply for less privileged inventors.

## Advice for Companies

As opposed to us doctors and innovators, industries seem to employ a large contingent of lawyers whose job is to ensure that the company pays as little as possible or nothing on the original invention. Therefore, they can take advantage, or even cheat us if their leaders do not take responsibility for how their company treats inventors. Even in 1993, a small company like SST had retained their own Chief Patent Counsel. He was much sharper than me in formulating contract law and knew how to apply maximum financial pressure. Indeed, it may very well be his job description in a company, but it is ultimately always the responsibility of the CEO and the Board-of-Directors to control the lawyers and ensure that inventors are treated in a fair, respectful and correct manner. When a company buys a patent, a license or a sublicense and signs an agreement, they also buy the scientific research history. In the TAVI case it took us more than 6 years of hard work and fighting to create the huge value of the invention and the strong patent. Therefore, the license is not just a piece of paper which can be sold to the next company without recognizing the innovators hard work.

Initially, the commercial value of an invention and a patent/license is low because nobody knows if it can be developed into a business success. Therefore, the initial payment to the inventor can also be low. In our case it was $20,000 in down payment which was fair at that time. When Heartport sold the license to PVT, the value had increased to $3 million plus 3.5% equity in PVT, and when Edwards purchased PVT for $125 million plus $30 million, the value of the license had increased significantly, but it was not reflected in royalty to the patent holders. When Edwards had developed the invention and started commercial sales, the value of the patent and license had increased dramatically with our assistance. Therefore, it should also have been shared with the inventors. The same was the case when Edwards cross-licensed the invention to Medtronic several years after the patent had expired. None of the two companies rectified the original flawed license agreement and shared any of the device's value with the inventors.

## Advice for Inventors and Companies

I hope my business story can be an inspiration and wake up call for new inventors, and for companies, too. Doctors are educated in natural and medical science, not in legal matters. We excel at coming up with new creative ideas and products which companies need and can benefit from by developing them beyond the early innovation stage. Inventors and companies simply must find a better way to work together in the future to develop new treatments and products. We need each other, and all good collaborations must be mutually beneficial. So far, the TAVI invention has been a disruptive clinical game changer which has generated great value for patients. It has created a major paradigm shift and has radically changed the way we treat patients with aortic stenosis worldwide. It has saved many thousand lives and it has rewarded several companies and investors with a multi-billion-dollar business, but the inventors and their academic institution where TAVI was invented did not gain anything near equivalent advantage from the success.

In the future, cooperation between academic inventors and companies should be transparent and made partly public to facilitate our academic freedom to teach and publish. Otherwise, academic inventors are tied hands, feet and mouth and cannot report the truth as they see it. Already back in 1993, SST's lawyers formulated confidentiality clauses and all the successive companies involved in the license and patents they forwarded new agreements with confidential clauses which we were required to sign. As doctors we hardly understood what it was all about. Our confidentially agreements with Edwards are life long, so the present business story about TAVI could never be told without breaking a few of the secret clauses in the agreement. For example, I am not allowed today to tell the truth that in 2007 we formed a partnership to defend our patent and I am not allowed to reveal how we assisted Edwards for many years in litigation in Europe and USA to make sure the Andersen patent prevailed and survived. Similarly, I am not allowed to disclose how we helped Edwards in USPTO meetings to successfully prolong the lifetime of the Andersen patent. Furthermore, I must not explain how our partnership with Edwards was manipulated so it suddenly ended prematurely and prevented us from receiving the huge compensation which we should have received according to the partnership agreement. However, such business information is crucial for new inventors to learn from and can only be told if we are not restricted by confidentiality clauses.

## Summary

When an academic inventor comes up with a new idea which has the potential to be disruptive and create a paradigm shift, he must be prepared to be met with skepticism, resistance and annoyance when he claims that the established rules no longer define the game. He will face ups and downs bringing his invention forward. Some of his colleagues will not believe him and sometimes even react with anger if it is their field he challenges. The response is widespread, even to the level of editors and review committees of scientific journals and even from powerful forces among his own peers and academic societies. At the same time, he must fight for his intellectual property, even when it appears to be futile. Industry must understand that the individual academic inventor does not live in the world of attorneys and contract and patent law like the companies do. Therefore, the responsibility falls to the company to establish a decent code-of-conduct which ensures that the inventor gets a fair treatment. This business ethos will serve companies, inventors and patients best.

## Author Contributions

The author conceived the paper and received substantial factual, historical and linguistic input from J. Michael Hasenkam and Frederic Joyce (see acknowledgements).

## Conflict of Interest

The author declares that the research was conducted in the absence of any commercial or financial relationships that could be construed as a potential conflict of interest.

## Publisher's Note

All claims expressed in this article are solely those of the authors and do not necessarily represent those of their affiliated organizations, or those of the publisher, the editors and the reviewers. Any product that may be evaluated in this article, or claim that may be made by its manufacturer, is not guaranteed or endorsed by the publisher.

## Abbreviations

BAV: balloon aortic valvuloplasty
FIA: first-in-animal
FIM: first-in-man
JACC: Journal of the American College of Cardiology
PTCA: percutaneous transluminal coronary angioplasty
TAVI: transcatheter aortic valve implantation
DTI: Danish Technology Institute
Edwards: Edwards Lifesciences
J&J: Johnson & Johnson
SST: Stanford Surgical Technologies
PVT: Percutaneous Valve Technologies.
